# LncRNA *CARMN* m6A demethylation by *ALKBH5* inhibits mutant *p53*‐driven tumour progression through *miR‐5683/FGF2*

**DOI:** 10.1002/ctm2.1777

**Published:** 2024-07-22

**Authors:** Nannan Liu, Xinxiu Jiang, Ge Zhang, Shuaiyu Long, Jiehan Li, Meimei Jiang, Guiyun Jia, Renyuan Sun, Lingling Zhang, Yingjie Zhang

**Affiliations:** ^1^ School of Biomedical Sciences Hunan University Changsha China; ^2^ Department of Laboratory Medicine The Third Xiangya Hospital Central South University Changsha China; ^3^ Department of Gastroenterology The First Affiliated Hospital of Zhengzhou University Zhengzhou China; ^4^ Hebei Provincial Mental Health Center Hebei Key Laboratory of Major Mental and Behavioral Disorders The Sixth Clinical Medical College of Hebei University Baoding Hebei China; ^5^ Department of Gastroenterology Huadong Hospital, Shanghai Medical College, Fudan University Shanghai P.R. China

**Keywords:** *ALKBH5*, *CARMN*, *miR‐5683*, mutant *TP53*, *FGF2*

## Abstract

N‐methyladenosine (m6A) represents a prevalent RNA modification observed in colorectal cancer. Despite its abundance, the biological implications of m6A methylation on the lncRNA *CARMN* remain elusive in colorectal cancer, especially for mutant *p53* gain‐of‐function. Here, we elucidate that *CARMN* exhibits diminished expression levels in colorectal cancer patients with mutant *p53*, attributed to its rich m6A methylation, which promotes cancer proliferation, invasion and metastasis in vitro and in vivo. Further investigation illustrates that *ALKBH5* acts as a direct demethylase of *CARMN*, targeting 477 methylation sites, thereby preserving *CARMN* expression. However, the interaction of mutant p53 with the *ALKBH5* promoter impedes its transcription, enhancing m6A methylation levels on *CARMN*. Subsequently, *YTHDF2/YTHDF3* recognise and degrade m6A‐modified *CARMN*. Concurrently, overexpressing *CARMN* significantly suppressed colorectal cancer progression in vitro and in vivo. Additionally, *miR‐5683* was identified as a direct downstream target of lncRNA *CARMN*, exerting an antitumour effect by cooperatively downregulating *FGF2* expression. Our findings revealed the regulator and functional mechanism of *CARMN* in colorectal cancer with mutant *p53*, potentially offering insights into demethylation‐based strategies for cancer diagnosis and therapy. The m6A methylation of *CARMN* that is prime for mutant *p53* gain‐of‐function‐induced malignant progression of colorectal cancer, identifying a promising approach for cancer therapy.

## Graphical abstract

1. *CARMN* was downregulated by mutant *p53* through ALKBH5.
2. Mutant *p53* transcriptionally decreased the expression of *ALKBH5* by binding to its promoter.
3. *CARMN* collaborates with *miR‐5683* to downregulate *FGF2* and induce autophagy.
4. CARMN suppressed colon cancer growth in vivo and could be used as a potential tumour inhibitor.

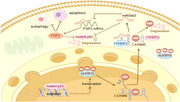

## 1 INTRODUCTION

Current research indicates that approximately 90% of the human genome is translated into RNA, but less than 2% of it contains protein‐coding genes.^1^, ^2^ Despite being characterised as ‘junk DNA’, more than 80% of the human and mouse genomes are translated into various RNA species, such as LncRNAs (Long noncoding RNAs), miRNAs, siRNAs and piRNAs.^3^ LncRNAs, characterised by their length of over 200 nucleotides and lack of discernible protein‐coding capacity, play pivotal roles at transcriptional, translational and posttranslational levels across diverse physiologic and pathologic conditions.^4^, ^5^ It is evident that although lacking extensive open reading frames, lncRNAs exert critical physiological functions as RNA molecules. The activity of lncRNAs is determined by the complicated interaction between RNA‐binding proteins and microRNA‐mediated pathways in cancer. Furthermore, the discovery and characterisation of lncRNAs in the human genome are rapidly expanding, underscoring the dynamic landscape of noncoding RNA biology.^6^

*CARMN* (cardiac mesoderm enhancer‐associated noncoding RNA) stands out as a highly abundant and evolutionarily conserved specific lncRNA in smooth muscle cells. Recent studies have unveiled *CARMN* as the host lncRNA for the MIR143/145 cluster, imparting crucial roles in cardiac and smooth muscle cell differentiation, as well as phenotypic regulation.^7^, ^8^, ^9^, ^10^, ^11^, ^12^, ^13^, ^14^ Notably, the early depletion of *CARMN* emerges as a crucial event driving VSMCs (Vascular smooth muscle cells) towards a pro‐atherogenic phenotype in vitro, while also hastening atherosclerosis progression in vivo.^15^ Silencing *CARMN* presents a promising therapeutic avenue for atherosclerosis, offering a novel target beyond conventional lipid reduction or anti‐inflammatory strategies, as it effectively curtails VSMCs (Vascular smooth muscle cells) proliferation in atherosclerotic plaques.^16^ However, investigations into the involvement of lncRNAs in the regulation of mutant p53 induced colorectal cancer remain sparse.

Recent studies have underscored the significant involvement of epigenetic modifications in tumour initiation and progression. N6‐ethyladenosine (m6A) stands out as the most prevalent epitranscriptomic alteration in eukaryotic cells, initially documented in the 1970s.^17^, ^18^, ^19^ The dynamics of m6A modifications are orchestrated by a dynamic and reversible process mediated by the m6A methyltransferase complex (MTC), comprising key components such as *METTL3* (methyltransferase‐like 3), *METTL14*, *WTAP* (Wilms tumour 1‐associated protein), while demethylation is facilitated by *ALKBH5* (AlkB homolog H5) and *FTO* (fat mass and obesity‐associated protein).^20^, ^21^, ^22^, ^23^, ^24^ Notably, *ALKBH5* has been implicated in tumour progression, particularly in breast cancer, where it sustains the stemness of cancer cells as a major demethylase of m6A alterations.^25^

Deletion of ALKBH5 has been linked to *p53* mutation, cytogenetic abnormalities, and decreased overall survival as well as event‐free survival in acute leukaemia. However, the role of *ALKBH5* in cancer appears contradictory based on recent studies.^26^, ^27^ Notably, it has been identified that the m6A demethylase‐encoding gene ALKBH5 has a polymorphism called rs8400 G > A, increasing susceptibility to neuroblastoma and shedding light on associated mechanisms. Aberrant regulation of ALKBH5 by miR‐5683, induced by this genetic variant, enhances neuroblastoma formation and progression through the ALKBH5‐SPP1 axis.^28^ Additionally, the stimulation of p53‐induced *ALKBH5* transcription regulated the m6A alterations in pancreatic cancer.^27^ Conversely, decreased *ALKBH5* expression was observed alongside inhibition of *p53* transcriptional activity or its knockdown in CSCs (nonsmall‐cell lung cancer‐derived cancer stem‐like cells).^29^ Nonetheless, the characteristics of *ALKBH5*‐mediated m6A alteration and its pathological significance in mutant p53‐induced colorectal cancer remain elusive.

Knockdown of *LINRIS* (Long Intergenic Noncoding RNA for IGF2BP2 Stability) led to decreased levels of *IGF2BP2* (insulin‐like growth factor 2 mRNA‐binding protein 2) in CRC cells, impacting MYC‐mediated glycolysis.^30^ *METTL3*‐mediated m6A medication of *THAP7‐AS1* increased its expression by the *IGF2BP1*‐dependent pathway.^31^ *FTO* demethylates the LncRNA *LINC00022* at the m6A position, promoting the formation of tumours in ESCC (esophageal squamous cell carcinoma) in vivo.^32^ Knockdown of *METTL14* significantly reduced the m6A level of *XIST* resulting in increased *XIST* expression.^33^ The N6‐methyladenosine modification by *METTL3* contributed to the upregulation of *LINC00958* by stabilising its RNA transcript.^34^ Functionally, *CARMN* was found to suppress bladder cancer proliferation via the *miR‐1275/AXIN2/Wnt/β‐catenin* pathway.^35^ *CARMN* enhanced cancer cell death by sponging *miR‐125a* to upregulate *p53* in endometrial carcinoma.^36^ Further investigation is warranted to elucidate the regulatory role of *CARMN* in colorectal cancer development, particularly in the context of mutant *p53*.

The *TP53* mutation plays a pivotal role as a target of genomic instability, contributing to accelerated tumour growth and diminished patient survival rate. LncRNAs have critical functions in colorectal cancer maintenance. However, it remains unclear whether mutant *p53* regulated lncRNAs implicated in colorectal cancer pathogenesis. This study identified lncRNA *CARMN* as a target of p53R273H specifically in colorectal cancer. Overexpression of *CARMN* attenuates colorectal cancer proliferation in the presence of mutant *p53*. Furthermore, m6A modification affects lncRNAs stability, yet its role in *TP53*‐mutant colorectal cancer remains elusive. The m6A demethylase *ALKBH5* exhibits low expression in mutant *p53* induced colorectal cancer. It was demonstrated that mutant *p53* binds to the *ALKBH5* promoter, suppressing its transcription and subsequent protein production. *CARMN* was identified as a major regulator in colorectal cancer, directly demethylated by *ALKBH5*, thereby acting as a suppressive modulator of colorectal cancer proliferation and differentiation. Additionally, *YTHDF2* and *YTHDF3* were downregulated in response to *CARMN* overexpression. The interplay between *CARMN* and *ALKBH5* promoted tumourigenesis in colorectal cancer patients via the *p53/ALKBH5/CARMN/miR‐5683* pathway. These findings illuminate the role of m6A methylation in colorectal cancer patients with *p53R273H* mutation.

## 2 MATERIALS AND METHODS

### 2.1 Cell lines and tissue treatment

Human colorectal cancer cell lines (HIEC‐6, FHC, HCT116, SW480, SW620, HT29) were authenticated by the International Cell Line Authentication Committee and obtained from ATCC. The cells were cultured in DMEM medium (Gibco) supplemented with 10% FBS at 37°C in a 5% CO_2_ atmosphere using Thermo Fisher Scientific incubators.

### 2.2 Data extraction and analysis

Data from RNA sequencing (RNA‐Seq), miRNA sequencing (miRNA‐Seq), masked somatic mutation, and clinical information of colon and rectum patients were obtained from The Cancer Genome Atlas (TCGA) (Genomic Data Commons Data Portal (GDC Data Portal), RRID:SCR_014514). The study included a total of 449 individuals with colon cancer and 94 with rectum cancer, comprising 212 mutant TP53 patients, and 237 wild‐type patients in the colon, as well as 68 mutant TP53 patients, and 26 wild‐type patients in the rectum. Level 4 reverse‐phase protein array data of patients were obtained from The Cancer Proteome Atlas (TCPA) (https://www.tcpaportal.org/). The expression data of LncRNAs, miRNAs, and mRNAs were analysed and processed using the DESeq2, RRID:SCR_000154 and EdgeR R packages. Volcano plots were generated using the ggpur and ggthems packages in R software, while survival curve plots were plotted using the survival and survminer packages in R software.

DemiRNAs targeted by DeLncRNAs were identified using the LncBase v3 online tool (https://diana.e‐ce.uth.gr/lncbasev3/). Potential mRNAs targeted by DEmiRNAs were predicted using the miRWalk database (miRWalk, RRID:SCR_016509, http://mirwalk.umm.uni‐heidelberg.de/). Protein–protein interaction (PPI) networks between the DemRNAs were constructed using the online tool STRING database (STRING, RRID:SCR_005223, http://string‐db.org/). The Cytoscape (RRID:SCR_003032, version 3.8.2) was employed to calculate the core value of DemRNAs in PPI networks and to analyse the lncRNA–miRNA–mRNA network.

### 2.3 Structure determination

To study the structure of *CARMN*, the secondary structure prediction of lncRNAs was performed using the website (http://rna.tbi.univie.ac.at/).

### 2.4 Western blotting

The collected cells were lysed on ice for 30 min using RIPA lysis buffer (Beyotime Institute of Biotechnology) supplemented with proteinase and phosphatase inhibitors (Selleck). Protein content was determined using a bicinchoninic acid (BCA) protein test kit (Thermo Fischer Scientific). Subsequently, proteins from each sample were transferred to PVDF membranes and blocked for 1 h at room temperature with 5% no‐fat milk. The PVDF membranes were then incubated overnight at 4°C in the antibody solution. Following incubation, the membranes were rinsed three times for 10 min with PBST and incubated for 1 h with secondary antibodies. After another three washes with PBST for 10 min, protein bands were visualised using an Ultra High Sensitivity ECL Kit (GLPBIO, Cat#GK10008) and detected with the Odyssey infrared imaging system (LICOR, Lincoln, NE).

### 2.5 Real‐time quantitative PCR

Total RNA was extracted from cancer cells using the RNA isolater Total RNA Extraction Reagent Kit (Vazyme). RNA quality was assessed using the NanoDrop ND‐1000, and RNA integrity was verified by conventional denaturing agarose electrophoresis. The cDNA synthesis was performed using 1 µg of total RNA with the Hiscript III All‐in‐one RT SuperMix Perfect for qPCR Kit (Vazyme). RT‐qPCR was conducted using the ChamQ Universal SYBR qPCR Master Mix Kit (Vazyme). Primer sequences used for gene amplification were provided in Table 1.

**TABLE 1:** 

gene	Forward primer	Reverse primer
FTO	TCGCATGGCAGCAAGCTAAA	GCACATTCCCTGACTCCACC
ALKBH5	AGTTCAGTCTTCTGCTCGCC	AGGAACTGTGGACATGGCAG
phlpp2	GTGCTCCACAAAAGGAGGGG	CAGCCGAGGTCAGGATTTGT
p21	TGTGGACCTGTCACTGTCTTG	GAACCTCTCATTCAACCGCCT
TNNT2	GCCCAATGGAGGAGTCCAAA	CCCACTTTTCCGCTCTGTCT
CD68	CTACTGGCAGAGAGCACTGG	GCTTCCCTGGACCTTGGTTT
CCL4L1	TGCCCCCACATTTGTCCTA	TAGCACGAGGAGAGACAGGA
CXCL13	TCAGCAGCCTCTCTCCAGT	TGGACAACCATTCCCACGG
IL6	GGTCCAGTTGCCTTCTCCCTG	GCCCATGCTACATTTGCCG
CXCL9	AGAAAGGGTCGCTGTTCCTG	GGGCTTGGGGCAAATTGTTT
CARMN	CAGAGCCGCCAGGTAAAACT	CAGGATGAGAGACACCGCTT
Actin	TGGCACCCAGCACAATGAA	CTAAGTCATAGTCCGCCTAGAAGCA

### 2.6 Fluorescence in situ hybridisation (FISH)

FAM‐labelled RNA probes targeting LncRNA CARMN were synthesised by Beijing Tsingke Biotech Co., Ltd (Beijing, China). Cells were plated on 24‐well plates and fixed with paraformaldehyde upon reaching the appropriate density. Subsequently, hybridisation was conducted by adding the LncRNA *CARMN* probe mix followed by incubation. After washing the cells, DAPI staining was performed in the dark, and the stained cells were examined under a fluorescence microscope (Zeiss LSM980).

### 2.7 Immunohistochemistry

Human colorectal tissues with mutant p53, wild‐type p53, and mouse tissues overexpressing luciferase, as well as LncRNA *CARMN*, were surgically excised and fixed in a 4% buffered paraformaldehyde solution overnight. Subsequently, the tissues were embedded in Paraffin wax and sectioned into 3 µm slices using the LEICA microtome system, which was preheated in warm water. The sections were then incubated in an incubator for at least 2 h. The EDTA‐Citrate Antigen Retrieval Solution (Beyotime Technology, China) was utilised for slide retrieval, and 1% hydrogen peroxide was applied to inhibit endogenous peroxidase activity. Following this, the slides were incubated overnight at 4°C with primary antibodies, including anti‐ALKBH5 (Proteintech Cat#16837‐1‐AP), anti‐FTO (Proteintech Cat#27226‐1‐AP), anti‐Ki67 (Proteintech Cat#27309‐1‐AP, RRID:AB_2756525), anti‐METTL3 (Proteintech Cat#15073‐1‐AP), anti‐m6A (ABclonal Cat#A19841), anti‐METTL14 (Proteintech Cat#26158‐1‐AP, RRID:AB_2800447), anti‐METTL16 (Proteintech Cat#19924‐1‐AP, RRID:AB_10639364), anti‐WTAP (Proteintech Cat#60188‐1‐Ig), anti‐p53 (Proteintech Cat#60283‐2‐Ig) and anti‐FGF2 (Proteintech Cat#11234‐1‐AP). After three washes in PBS, the slices were incubated with the appropriate secondary antibodies for 1 h at room temperature. Pictures were acquired and analysed using a Nikon Eclipse Ti2‐U microscope.

### 2.8 Chromatin immunoprecipitation

The chromatin immunoprecipitation (ChIP) experiment was conducted using specified regents and protocols provided by the ChIP Assay Kit (Beyotime Institute of Biotechnology). Colorectal cells were cross‐linked with formaldehyde for 10 min, followed by 5 min treatment with glycine solution at room temperature. Subsequently, the cells were placed on ice and lysed using SDS lysis buffer. Genomic DNA was sonicated in an ultrasonic breaker machine to yield DNA fragments 200−1000 bp. Cross‐links between protein and genomic DNA were removed by treating the cell lysates with NaCl at 65°C for 4 h. Immunoprecipitations were performed overnight at 4°C using a p53 antibody (Proteintech Cat#60283‐2‐Ig), as previously described.^37^ Following immunoprecipitation, the DNA‐protein complexes were washed three times at 4°C. The products for PCR detection were obtained after DNA purification using the universal DNA purification kit (Tiangen). Table 2 presents the primer sequence for ALKBH5 from the three predicted locations utilised in this investigation.

**TABLE 2:** 

gene	Forward primer	Reverse primer
p53‐A5‐site1	ACTGCCTGATTGACACGCAT	CCTTTGGCGCTTCCACTTCT
p53‐A5‐site2	TGGCGGTTCCCTGGTGAATG	TCCGCGCGCTACGGG
p53‐A5‐site3	CAATATGAGCGCACCCCTGTAGA	GACAACGGGGCTTCTTCCTCC

### 2.9 RNA‐binding protein immunoprecipitation (RIP) assay

Colorectal cancer cells were seeded in culture plates for 24 h prior to cotransfection with GFP‐CARMN, and a vector using Lipofectamine 2000. After 48 h, RNA immunoprecipitation was performed using antibodies against FTO, METTL3 and ALKBH5 from the EZ‐Magna RIP™ Kit (Millipore). Subsequently, RT‐PCR was conducted on pure RNA complexes, and coisolated RNA‐binding proteins were identified by real‐time quantitative PCR.

### 2.10 M6A‐RNA immunoprecipitation (MeRIP) assay

Total RNA was collected from SW480 cells transfected with plasmids overexpressing or downregulating *ALKBH5*, or from those transfected with an empty vector control. Genomic DNA was eliminated using DNase (Vazyme). Subsequently, mRNA was purified, and then fragmented. The resulting fragments underwent immunoprecipitation with an m6A antibody, employing the EpiQuik™ CUT&RUN m6A RNA Enrichment Kit (EpiGentek Group, Cat#P‐9008).

### 2.11 Luciferase reporter assay

Colorectal cancer cells were plated in 24‐well plates and incubated for 24 h before cotransfection with the luciferase reporter vector, and the Renilla vector. The luciferase reporter, the Renilla luciferase construct, and either miR‐5683 control, miR‐5683 mimics, or miR‐5683 inhibitors were transfected into SW480 cells using Lipofectamine 2000 (Invitrogen). Following 48 h of transfection, the ratios of firefly and Renilla luciferase activities were measured using the Dual‐Lumi™ Luciferase Reporter Gene Assay Kit (Beyotime Institute of Biotechnology). Subsequently, m6A‐modified mRNA enrichment was assessed via qRT‐PCR.

### 2.12 Nuclear/cytoplasmic isolation

Thermo Fisher's NE‐PER™ Nuclear and Cytoplasmic Extraction Reagents (Cat#78835) were used to isolate nuclear/cytoplasmic materials following the manufacturer's protocol. Subsequently, cytoplasmic and nuclear fractions were isolated for RNA extraction. Human β‐actin and U6 were respectively utilised as references for cytoplasmic and nuclear RNA.

### 2.13 RNA pull‐down

The SW480 cell lysates were incubated with Biotin‐labelled probes of CARMN provided by Tsingke (Beijing, China), followed by precipitation using streptavidin beads (Thermo Scientific Pierce). Subsequently, Western blot assay was performed on the precipitated ALKBH5 protein.

### 2.14 Cell viability and colony formation assay

Colorectal cancer cells were cultured in 96‐well plates at a density of 5 × 10^3^ cells per well. Cell viability was assessed using the CCK‐8 assay. Each assay was performed in triplicate. Additionally, colorectal cancer cells were seeded at a density of 1 × 10^3^ cells per well in six‐well plates. Following 2 weeks of incubation, colonies were fixed and stained with a 0.1% solution of Crystal Violet (Beijing Solarbio Science & Technology). Subsequently, the number of colonies was counted.

### 2.15 Cell flow cytometry

The number of labelled cells was quantified using a BD FACS Aria III flow cytometer (Catalog#: BD FACS Aria ™ III, BD Biosciences, Hercules, CA, USA).

### 2.16 CUT&Tag analysis

Cleavage Under Targets and Tagmentation (CUT&Tag) represents a novel approach for scrutinising the interaction between proteins and DNA fragments. This investigation applied the CUT&Tag technique to probe the potential downstream regulatory role of *p53* on the *ALKBH5* gene, along with identifying the specific binding site of p53 on the *ALKBH5* promoter in sw480 cells with knockdown mutant p53R273H. Subsequent to cell harvest, nuclear isolation and purification were conducted, followed by an overnight incubation of a p53 primary antibody at 4°C. The ensuing day involved a 30 min incubation of the secondary antibody with the hyperactive protein A/G‐Tn5 transpose to yield fragmented DNA. Subsequent purification and amplification of the DNA library were performed for sequencing on the IIIumina NovaSeq 6000 platform. Data analysis of the CUT&Tag results began with an assessment of raw data quality and the removal of substandard data using FastQC software. Furthermore, the IGV too was utilised to convert raw BAM files into bigwig files, thereby facilitating the visualisation of read count data.

### 2.17 Xenograft studies

Six‐ to eight‐week‐old female nude mice were inoculated with 1×10^6^ cells (control and Overexpressed *CARMN* SW480 cells) for each flank of five mice per group. Using a lentiviral system, SW480 cells were stably transduced with luciferase‐labelled *CARMN* overexpression plasmids using a lentiviral system prior to injection into mice. Metastasis was evaluated by intraperitoneal injection of D‐luciferin sodium followed by imaging using a small animal imager (IVIS SPECTRUM CT). Additionally, the mice were measured the weight every 2 days to evaluate treatment effects. Empty vector and OE‐CARMN xenografts were established and monitored for 14 days.

### 2.18 Statistical analysis

The sample sizes were determined based on pilot studies and our previous experience with comparable investigations. The data were graphically plotted using the R package and the GraphPad Prism (RRID:SCR_002798) version 6.02 software. The Kaplan–Meier estimation was used to compare patient survival curves. The two‐tailed Student's *t*‐tests and two‐way ANOVA were utilised to analyse group differences. The *p*‐value of less than .05 was considered statistically significant for two‐sided tests.

### 2.19 Data availability

All data necessary to evaluate the conclusions of this paper are provided in the paper and/or the Supplementary Materials. The datasets utilised and/or analysed during the current study are available from the corresponding author upon reasonable request.

## 3 RESULTS

### 3.1 CARMN was downregulated by mutant p53 through ALKBH5

**FIGURE 1:**
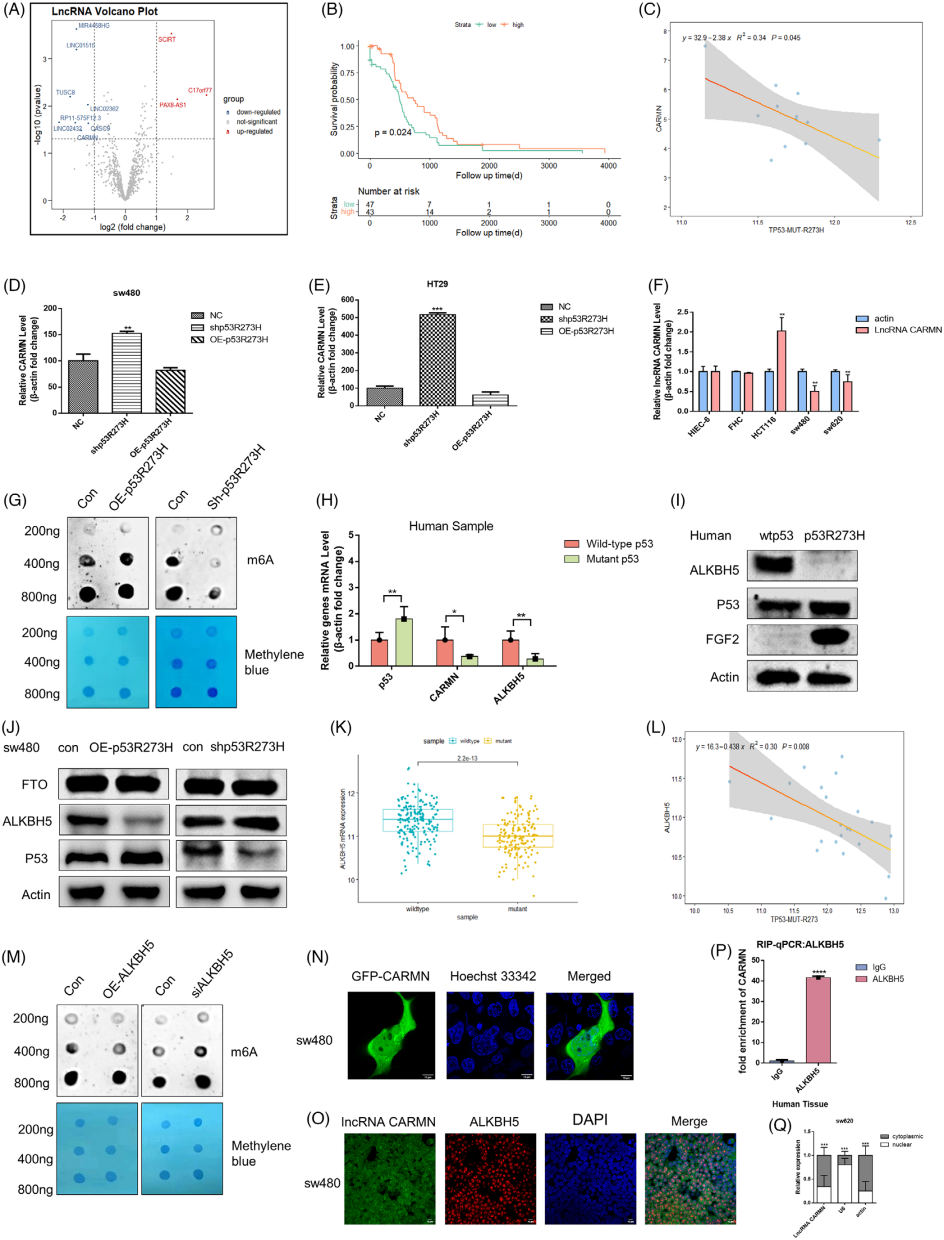
Downregulation of LncRNA *CARMN* is associated with high methylation levels in colorectal cancer with mutant *p53*. (A) A volcano plot displayed the significantly expressed LncRNAs between *TP53* mutant and wild‐type patients in CRC. (B) The plot of the survival curve showed the low expression of *CARMN* led to a poor prognosis in advanced colorectal patients with mutant *p53* and wild‐type *p53*. (C) The correlation between the demethylase LncRNA *CARMN* and mutant p53R273H was analysed and visualised using corrplot package with colon cancer data retrieved from the TCGA database. (D, E) The qPCR assays were employed to assess *CARMN* RNA levels in SW480 and HT29 cells transfected with human empty non‐p53 construct vector with GFP (NC), sh‐p53R273H or OE‐p53R273H, respectively. (F) Relative expression of LncRNA *CARMN* in the normal colorectal cancer cells (HIEC‐6, FHC) and other different colorectal cancer cell lines (HCT116, SW480, SW620). The scale bar in 10 µM, *p < 0.05 and **p < 0.01 as indicated. (G, M) m6A dot blot assays of SW480 cells with knockdown or overexpression of *p53*‐R273H (G) or *ALKBH5* (M) were designed to measure the m6A level from total RNA diluted with 200, 400 and 800 ng, methylene blue (MB) staining worked as a loading control. (H, I) The mRNA and protein expression of *ALKBH5* in colon cancer patients with mutant *p53*. (J) The relative protein level of *ALKBH5* in SW480 cells transfected with sh‐*p53R273H* or OE‐*p53R273H*. (K) The mRNA expression levels of the demethylase *ALKBH5* were compared between mutant and wild‐type *p53* using R Studio with data sourced from the TCGA database. (L) The correlation between the demethylase ALKBH5 and mutant p53R273H was analysed and visualised using corrplot package with colon cancer data retrieved from the TCGA database. (P) Human colorectal tissue samples containing mutant p53 were utilised to investigate the correlation between CARMN and ALKBH5 through the RIP assay, employing the ALKBH5 antibody. (N) The location of *CARMN* was imaged by confocal microscopy in SW480 transfected with GFP‐*CARMN*. (O) Combined immunofluorescence obtained from DAPI (blue) and RNA‐FISH analysis of LncRNA *CARMN* (green), and *ALKBH5* (red) in SW480 cells. (Q) The expression of *CARMN* in the subcellular fractions of colorectal cancer with mutant *p53* was detected by RT‐PCR. *U6* and *actin* were utilised as nuclear and cytoplasmic markers, respectively.

According to our previous finding, mutant p53 enhances colon cancer malignant growth and immune evasion by the *PHLPP2/AKT/PD‐L1* pathway.^37^ To investigate the expression of LncRNAs in colorectal cancer with mutant *p53*, *p53* missense mutation profile data was obtained from the Cancer Genome Atlas using the R maftools program (TCGA‐COAD dataset). For these samples, *CARMN* expression was downregulated with a significant difference. The volcano plot of the lncRNAs expression signal depicted the landscape of colon cancer samples with mutant p53R273H (212) and wild‐type p53 (237) based on clinical characteristics (Figure 1A). For these samples, *CARMN* expression exhibited a significant downregulation in these samples with mutant p53R273H. Next, we wonder to investigate whether *CARMN* expression affects colorectal cancer with mutant p53 patients’ clinical progression. Kaplan–Meier analysis showed that downregulated CARMN expression was related to worse survival probability in later periods’ patients of 90 colorectal samples with mutant p53R273H, compared to upregulated expression of *CARMN* (Figure 1B). The second structure of *CARMN* was displayed (Figure S1A). To confirm the coding transcripts from the non‐coding transcript, the Coding‐potential calculator coding potential assessment tool showed *CARMN* has a very weak protein‐coding potential (Figure S1B). This study investigated the correlation between mutant p53R273H and CARMN (*p* = .045), revealing a notable negative correlation with a *p*‐value below .05 (Figure 1C). Additionally, the correlation of other forms of mutant p53, such as R175H, R273C, R248Q, R282W, with lncRNA CARMN was detected. It was observed that R175H (*p* = .162), R273C (*p* = .534), R248Q (*p* = .788) and R282W (*p* = .847) had no significant correlation with lncRNA CARMN, as indicated by *p*‐values exceeding .5 (Figure S1H‐K). Simultaneously, plasmids contained various p53 mutation sites were constructed and transfected into p53^−/−^ cells to detect the CARMN expression levels. It indicated that a notable difference in CARMN expression with the TP53R273H mutation compare to other mutation sites in Figure S1U. These results indicated that the correlation between other mutant p53 variants and CARMN was not significance except for mutant p53R273H. In addition, the volcano plots elucidated the relationship between mutant p53 samples from colon cancer in the TCGA database and long non‐coding RNAs (LncRNAs) (Figure S1L‐O). The human colorectal cancer cell lines (SW480, SW620, and HT29) harboured a missense mutation at codon 273 (p53R273H) in our previous research.^37^ Then, to investigate the regulatory role of mutant p53 on *CARMN*, two cell lines (SW480, HT29) were transfected with either an empty construct vector with GFP, sh‐p53R273H, or oe‐p53R273H, respectively. Fluorescence microscopy confirmed the presence of GFP fluorescence in transfected cells in Figure 1D and E. Notably, knockdown of p53R273H led to an increase in *CARMN* expression compared to the control group. Conversely, overexpression of p53R273H resulted in a reduction of *CARMN* expression levels. Besides, the expression of LncRNA *CARMN* was measured in normal colon epithelial cell lines (HIEC‐6 and FHC) and several colon cancer cells (HCT116, SW480, SW620), and the results revealed that *CARMN* expression was significantly reduced in the colon cell lines of mutant p53 (Figure 1F).

The function of N6‐methyladenosine (m6A) in *TP53*‐mutant colorectal cancer is unknown, which is crucial for mRNA stability, translation and splicing. To study the role of m6A modification in colorectal cancer cells with mutant *p53*, the results of an m6A dot blot assay indicated that global m6A RNA levels of *p53* mutation were clearly increased (Figure 1G). Furthermore, it was shown that the effect of *p53* expression and m6A methylation in SW480 cells (Figure S1E). Additionally, the mRNA and protein levels of *p53, CARMN, ALKBH5* and *FGF2* were assessed in human colorectal cancer tissues with both wild‐type and mutant p53 (Figures 1H and I and 6H). As a result, the expression of *CARMN* and *ALKBH5* is significantly downregulated while *FGF2* is marked upregulated in human colorectal cancer with mutant *p53*. Meantime, the expression of mutant *p53* is much higher than that of the wild type *p53* in human cancer samples. Similarly, the protein levels of *ALKBH5* were lower, with higher‐level mutant *p53* expression in colorectal cancer cells (Figure 1J). In addition, the clinical findings of *ALKBH5*, *FTO*, and *METTL3* mRNA expression were summarised in colorectal patients of wild‐type and mutant *p53* (Figures 1K and L and S1F and G). However, the trends of *FTO* (Figure S1F) and *METTL3* (Figure S1G) mRNA expression with *p53* showed negligible statistical significance in the samples analysed. Besides, Figure 1J indicated that the protein expression of *FTO* did not significantly affect the abundance of colorectal cancer with mutant p53. The correlation analysis of p53R175H, p53R248Q and *ALKBH5* was performed using the R language. The data of human colorectal cancer samples were downloaded from TCGA and analysed by using R language. The results of Figures 1L and S1P showed that both mutant p53R273H (Figure 1L) and p53R175H (Figure S1P) have strong negative correlation with *ALKBH5* (*p* < .05), while mutant p53R248Q (Figure S1Q) does not (*p* > .05). On the contrary, the volcanic map gives us different results that mutant p53R175H (Figure S1R) has no significant correlation with *ALKBH5*, nor does mutant p53R248Q (Figure S1S). Subsequently, direct interaction between *ALKBH5* and *CARMN* was confirmed via RIP assay in both human tissue and mouse xenograft tumours. ALKBH5‐RIP assay identified lncRNA *CARMN* as a direct target of *ALKBH5* in human tissue and mouse xenograft tumours (Figures 1P and 8H). Next, the influence of *ALKBH5* on m6A methylation was studied by using the dot blot assay. As a result, the m6A methylation levels of RNA significantly declined after overexpressing *ALKBH5*, while increased evidently in the *ALKBH5* knocking down group (Figure 1M). Concurrently, IHC assays were performed in mutant *p53R175H* of colorectal cancer samples. The analysis of *Ki67* and *m6A* showed that strong signals specifically surrounded the mutant *p53* tissue. Besides, *ALKBH5, FTO, METTL3, METTL14, METTL16* and *WTAP* were detected in a section of colorectal cancer tissue (Figure S1T). To gain insight into where *CARMN* was distributed throughout the subcellular environment, we observed the presence of *CARMN* by fluorescence imaging in a single cell (Figure 1N). Furthermore, bioinformatics prediction and the nuclear/cytoplasmic RNA separation test findings revealed that *CARMN* was predominantly found in the cytoplasmic (Figures 1O and Q and S1C and D). Combined immunofluorescence and FISH assays confirmed this phenomenon (Figure 1O).

These results indicated that mutant *p53* downregulates *ALKBH5* and *CARMN* expression. Further investigating the specific molecular mechanisms could help inhibit the progression of colorectal cancer with mutant *p53*.

### 3.2 Mutant *p53* transcriptionally decreased the expression of *ALKBH5* by binding to its promoter

**FIGURE 2:**
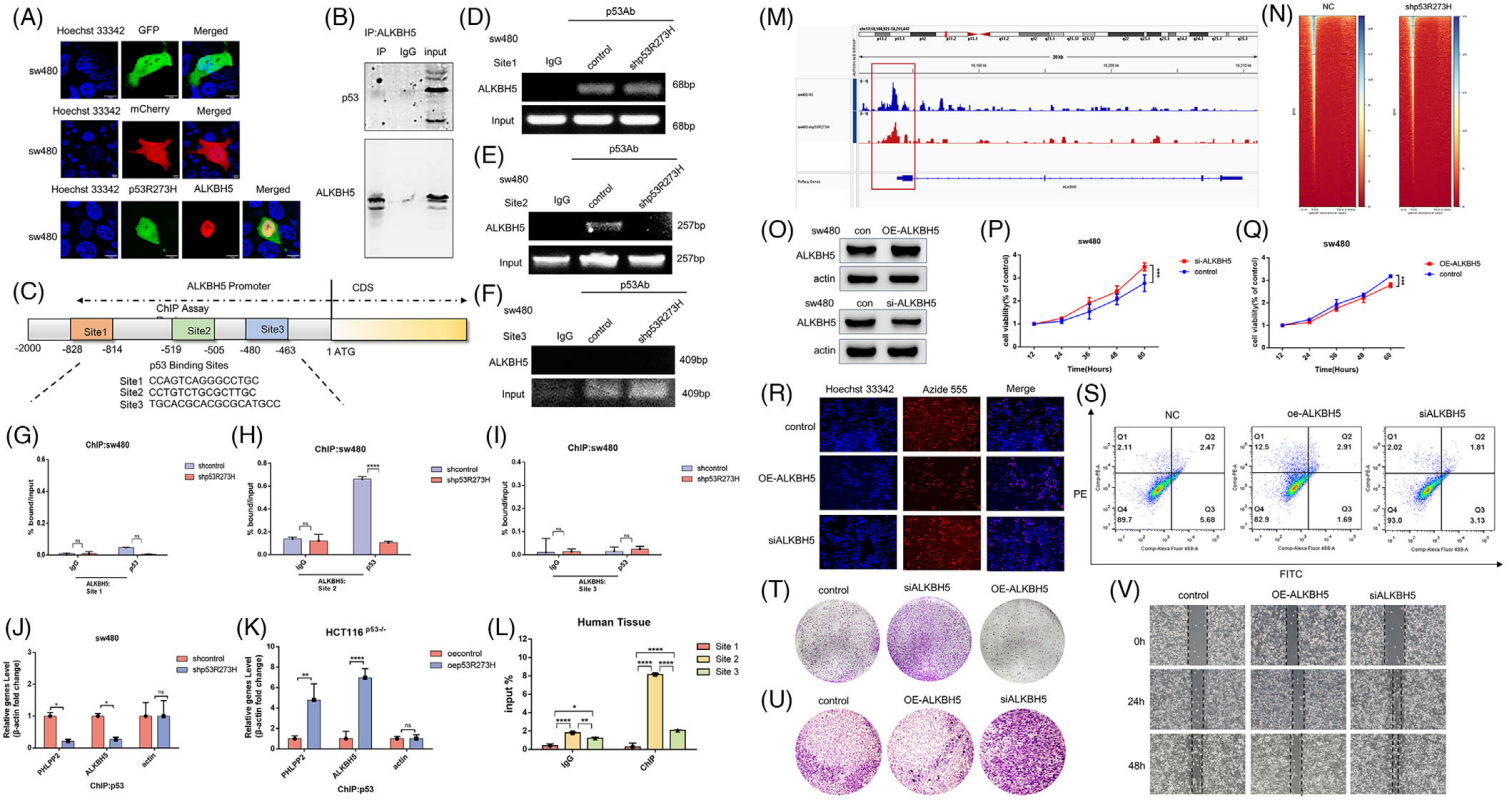
Mutant *p53* inhibits the transcription of *ALKBH5* to promote the malignant proliferation of colorectal cancer with mutant *p53*. (A) The live SW480 cells transfected with GFP‐*p53R273H* and mCherry‐*ALKBH5* were imaged by confocal microscopy. (B) Co‐immunoprecipitation (Co‐IP) analysis of the interaction between *ALKBH5* and mutant *p53* in SW480 cells. (C) Three potential mutant‐*p53* binding sites on the *ALKBH5* promoter were shown in the diagram. (D–I) A specific binding site was demonstrated by ChIP‐qPCR analysis. DNA fragments were precipitated using either a p53‐specific antibody or an IgG antibody. The input was employed as internal positive controls, whereas IgG served as an internal negative control. (J, K) ChIP‐qPCR assays further confirmed the direct binding of mutant p53 to the promoters of ALKBH5, PHLPP2 and p21 in SW480 cells with mutant p53 knockdown (J), as well as in HCT116 p53^−/−^ cells with mutant p53 overexpression (K). (L) ChIP‐qPCR analysis conducted on human tissue confirmed three sites of mutant p53 binding to the ALKBH5 promoter, with IgG served as an internal negative control. (M) The CUT&Tag assay confirmed that mutant p53 directly bound to ALKBH5 promoter region. Compared to the untreated control group, ALKBH5 promoter signal was significantly decreased in sw480 cells with knockdown of mutant p53R273H. The red box indicated the binding site of mutant p53 on ALKBH5 promoter. Characteristic signal peaks in this region were shown for both the control group (upper) and knockdown p53R273H (lower). (N) Profile plots and heatmaps were displayed the mutant p53 signal across the gene bodies or transcription start sites (TSS) of ALKBH5 RT&Tag‐enriched transcripts based on varying levels of p53 CUT&Tag signal over their gene bodies. The heatmaps were arranged in descending order of CUT&Tag signal strength. (O) The relative protein level of ALKBH5 in SW480 cells transfected with OE‐*ALKBH5* or si‐*ALKBH5*. (P–T) The CCK8 assays (P, Q), the EdU assays (R) and colony formation assays (T) were obtained to measure the effect on SW480 cells transfected with OE‐*ALKBH5* or si‐*ALKBH5*. (S) The SW480 cells were treated with overexpression of *ALKBH5* or knockdown of *ALKBH5* to induce apoptosis. The cells were stained with Annexin V‐iFluor 488 and Propidium Iodide. Apoptosis cells can be observed in the bottom right quadrant. (U, V) The transwell migration assays (U) and wound‐healing assays (V) were applied to compare the cell proliferation or migration ability in SW480 cells transfected with OE‐*ALKBH5* or si‐*ALKBH5*.

Studies have shown that *ALKBH5* was revealed to be positively correlated with wild‐type *p53* in lung cancer by the Gene Expression Profiling Interactive Analysis (GEPIA) web tool. Further, *p53* affected *ALKBH5* transcription to influence the global m6A methylation level.^29^ The stimulation of *ALKBH5* transcription by *p53* operated as a feedback loop to control the m6A modification in pancreatic cancer.^27^ However, the relation between mutant *p53* and *ALKBH5* has not been reported. The fluorescence imaging of GFP‐*p53R273H* and mCherry‐*ALKBH5* was performed to study their subcellular localisation in SW480 cells. As shown in Figure 2A, the colocalisation of GFP‐*p53R273H* and mCherry‐*ALKBH5* in the same cells was observed. To determine the relation of *ALKBH5* and *p53*, the Co‐IP assay showed that mutant *p53* did not directly bind to *ALKBH5* (Figure 2B). To investigate if mutant *p53* could bind to the promoter of *ALKBH5* and pinpoint the exact binding sites, three possible binding sites were generated by bioinformatics analysis (Figure 2C). Then, we detected the activity of different regions of the *ALKBH5* promoter. The ChIP assay indicates that the activation of the ALKBH5 promoter was impeded by the binding of mutant p53 to the promoter region of ALKBH5. Conversely, ALKBH5 promoter inhibition was totally reactivated when the mutant p53 was knocked down, suggesting a direct binding between mutant p53 and this specific genomic region (Figure 2D‐I). The results showed clearly that after knocking down the expression of mutant p53, the binding with site 2 decreased significantly, while the binding with site 1 or site 3 had no change (Figure 2G‐I). These results demonstrated that site 2 is the binding site of mutant p53 on ALKBH5 promoter.

Our recent study has shown that mutant p53 directly binds to the promoter of PHLPP2 and inhibits its transcription.^37^ In order to further confirm the regulation on ALKBH5 by mutant p53, PHLPP2 and actin were conducted in our present study as the positive and negative control. As a result, overexpressing p53R273H markedly increased the binding between mutant p53 and promoters of PHLPP2 and ALKBH5 (Figure 2K). Conversely, the deletion of p53R273H led to a decrease of these bindings (Figure 2J). While actin was not influenced by the change of mutant p53 (Figure 2J and K). The study was further validated in vivo. It was revealed that mutant p53 binds to the promoter of ALKBH5, inhibiting its transcription in human tissue and mouse xenograft tumours, elucidating the interplay between mutant p53 and ALKBH5 (Figures 2L and 8G). As a result, p53R273H activated the proximal −519 bp region of *ALKBH5*. We identified mutant p53 binding site in this region (Figure 2E, H and L). Additionally, as a transcription factor, mutant p53's binding motif on the promoter of *ALKBH5* was shown (Figure S2A and B). P53 has been shown to be transcriptionally activated on the ALKBH5 by binding to its promoter in pancreatic cancer, as supported by genome analysis, microarray verification, luciferase analysis, and bioinformatics prediction.^27^ Furthermore, the online tool Gene Expression Profiling Interactive Analysis (GEPIA) revealed a favourable correlation between ALKBH5 and p53 in lung cancer. It has been demonstrated that p53 transcriptionally regulated ALKBH5, thereby modulating the global m6A methylation level.^29^ To investigate the potential regulatory role of wild‐type p53 on the ALKBH5 promoter, a chromatin immunoprecipitation (ChIP) assay was performed in HCT116 cells. The findings demonstrated that wild‐type p53 could not directly interact with the ALKBH5 promoter in site 2 to suppress its transcription (Figure S2C).

As the highest rate mutant among all tumours,^38^ mutant p53R175H was picked up for the next functional investigation. Through genomic analysis, three binding domains for the mutant p53R175H protein were predicted in the ALKBH5 promoter, providing insights into the molecular mechanisms influenced by this mutant variant. To further assess whether p53R175H binds to the promoter of ALKBH5, the p53R175H plasmid was transfected into HCT116 p53^−/−^ cells, followed with ChIP and qPCR analyses. As shown below (Figure S2D‐F), mutant p53R175H has a direct interaction with ALKBH5 promoter, predominantly on site 2. The CUT&Tag assay was employed to investigate the direct regulation of *ALKBH5* by mutant *p53* in sw480 cells, aiming to provide further elucidation. Colorectal cancer cells were harvested and subjected to sequential incubation with a primary *p53* antibody, secondary antibody and protein A/G‐Tn5 transposase for the construction of a DNA library suitable for high‐throughput sequencing. The obtained results confirmed the direct binding of mutant *p53* to the promoter region of *ALKBH5*, with the binding site being located at the transcription start site (TSS). The peak signal of *ALKBH5* promoter was significantly lower in mutant p53R273H knockdown cells than that in the untreated group (Figure 2M and N). These findings provided the compelling evidence that mutant p53 transcriptionally suppressed *ALKBH5* expression by directly binding to *ALKBH5* promoter region.

To detect the function of *ALKBH5* in our system, cell viability was analysed by cell counting kit‐8 at 12, 24, 36, 48 and 60 h, respectively (Figure 2O‐Q). Consistent with a recent report,^27^ knocking down *ALKBH5* significantly increased the proliferation and migration rate, while contrary results were observed when *ALKBH5* was overexpressed in SW480 cells (Figure 2R and T). Next, flow cytometry was used to identify apoptosis in SW480 cells with overexpression of *ALKBH5* or knockdown of *ALKBH5*, respectively (Figure 2S). Besides, it revealed that depletion of *ALKBH5* induced cell cycle arrest at the S phase (Figure S2G). The cell scratch and migration tests showed that after 24 and 48 h of culture, the scratch distance and migration rate of SW480 cells decreased with knocking down *ALKBH5* (Figure 2U and V). Taken together, these findings suggest that mutant p53 is directly bound to the promoter of the *ALKBH5* region to inhibit its transcription, downregulated *ALKBH5* protein level, and accelerated colorectal cancer progression.

### 3.3 *ALKBH5* demethylated m6A *CARMN* and maintained its expression level by *YTHDF2*

**FIGURE 3:**
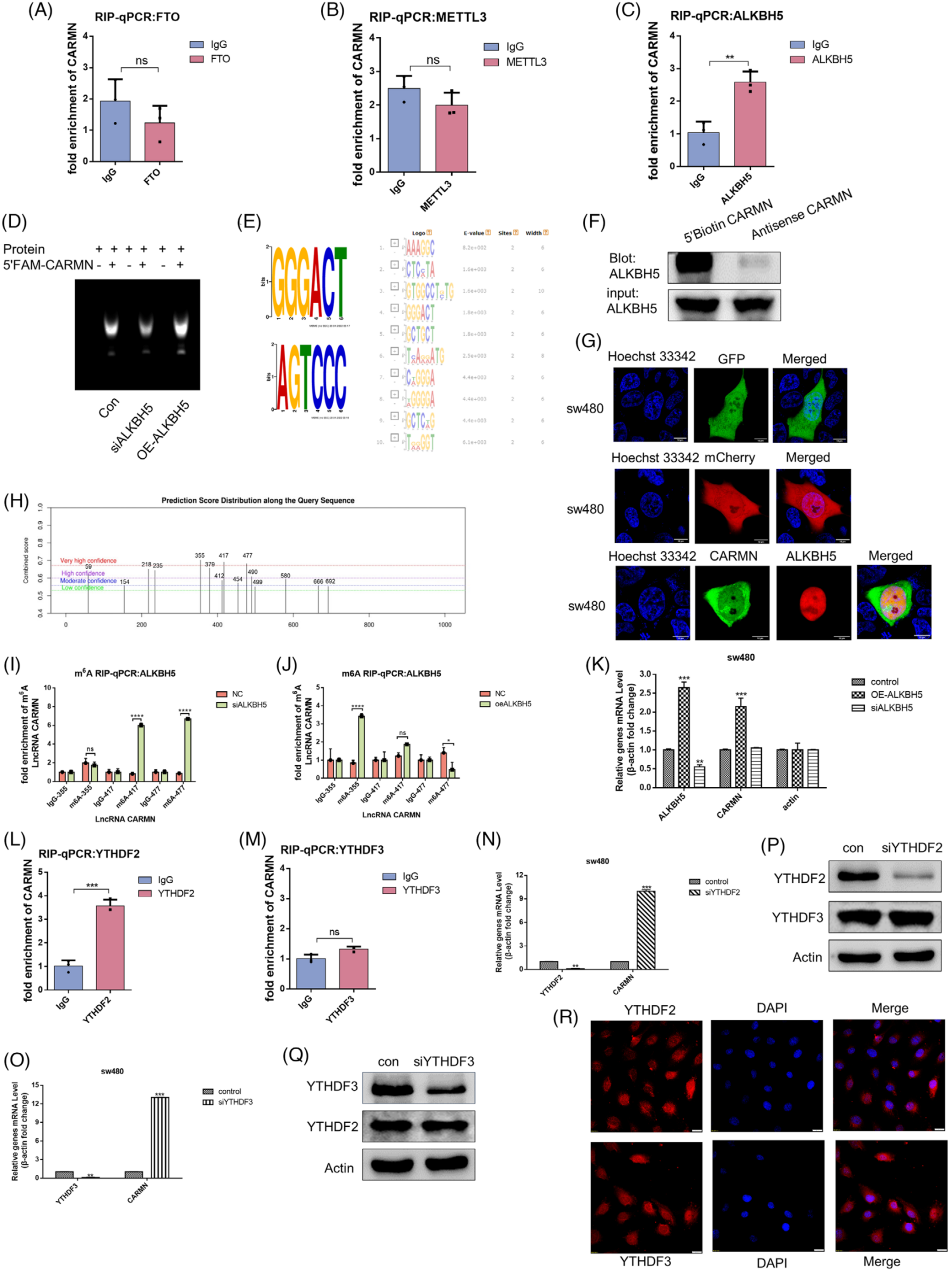
*ALKBH5* combines with LncRNA *CARMN* to remove its methylation modification, thereby regulating its expression. (A–C) RIP assays confirmed the association between *CARMN* and *FTO, METTL3* and *ALKBH5* in SW480 cells. (D) The concentration of ALKBH5 protein was mixed with FAM‐labelled *CARMN*. (E) Motif analysis was made by the online tool DREME to identify ‘ATGCC’ as the m6A consensus motif of *ALKBH5*. (F) Proteins obtained from the RNA pull‐down experiment were used to measure the quantification of *ALKBK5* in SW480 cells transfected with Biotin labelled *ALKBH5*. (G) The colocalisation of *CARMN* and *ALKBH5* was observed by confocal microscopy in SW480 transfected with GFP‐*CARMN* and mCherry‐*ALKBH5*. (H) The online tool SRAMP was used to analyse methylation modification sites of *CARMN*. (I, J) MeRIP assays interpreted the m6A sites in which *ALKBH5* combined with *CARMN* in SW480 cells transfected with OE‐*ALKBH5* or si‐*ALKBH5*. (K) The qPCR assays were used to analyse *CARMN* and *ALKBH5* RNA levels in SW480 cells transfected with OE‐*ALKBH5* or si‐*ALKBH5*. (L, M) The RIP assays displayed the reader, which could combined with the *CARMN* in SW480 cells by *YTHDF2* and *YTHDF3* antibodies. (N–Q) The expression of *CARMN* in SW480 or HT29 cells transfected with si*YTHDF2* or si*YTHDF3*. (R) The colocalisation of *YTHDF2* and *YTHDF3* with the nucleus was observed by Immunofluorescence in SW480 cells.

To study the relationship of *CARMN* with *ALKBH5* in colorectal cancer with mutant *p53*, an RNA immunoprecipitation (RIP) assay was performed and the results revealed that *CARMN* interacted with *ALKBH5* in SW480 cells (Figure 3A‐C). Further, the EMSA (electrophoretic mobility shift assay) analysis showed that FAM‐labelled *CARMN* oligos interacted with ALKBH5 proteins (Figures 3D and S3A). Additionally, the consensus motif search yielded the GGACT sequence, which has been identified as a consensus methylation motif by others.^39^, ^40^ A consensus motif was discovered, which was relatively obvious in the CDS of *ALKBH5* (https://meme‐suite.org/meme/tools/meme) (Figure 3E). According to the RNA pull‐down test, the binding between *ALKBH5* and biotin‐labelled *CARMN* decreased in the antisense of *CARMN* (Figure 3F). To visualise *CARMN‐ALKBH5* interaction, GFP‐*CARMN*, and mCherry‐*ALKBH5* were transfected in SW480 cells and their colocalisation was observed (Figure 3G). Importantly, the potential specific m6A site of *CARMN* that was implicated by *ALKBH5* was predicted using the SRAMP website (http://www.cuilab.cn/sramp/), and three candidates of m6A modification sites with high confidence were obtained (Figure 3H). The secondary structure of CARMN was depicted, and three potential m6A modification sites were predicted. Subsequently, m6A RIP assay validation revealed that the 477 m6A site exhibited the strongest functional activity (Figures 3I and J and S3B).

Further, overexpressed or knocked down *ALKBH5* increased or decreased the amounts of *CARMN* respectively in SW480 cells (Figure 3K). Studies have shown that YTHDF proteins may influence essential biological processes connected to m6A RNA methylation.^41^, ^42^ To investigate whether *YTHDFs* were involved in the m6A modification of *CARMN*, an RNA immunoprecipitation (RIP) assay was performed and the results revealed that an abundance of *CARMN* was presented in the complex pulled down by *YTHDF2* and *YTHDF3* (Figure 3L and M ). It indicated that *CARMN* interacted with *YTHDF2* and *YTHDF3*. Then, the effect of *YTHDF2* and *YTHDF3* on the stability of *CARMN* was tested. As shown in Figure 3N‐Q, knocking down *YTHDF2* or *YTHDF3* significantly increased *CARMN* expression. Additionally, the ALKBH5 protein was exclusively detected in the nucleus fractions, and *YTHDF2* and *YTHDF3* were localised in the cytoplasmic fractions (Figure S3C). Furthermore, the immunofluorescence detection showed that *YTHDF2* and *YTHDF3* gave us an identical result (Figure 3R). In summary, the m6A modification of *CARMN* was demethylated by *ALKBH5* and then bound to *YTHDF2* and *YTHDF3* in cytoplasmic fractions.

### 3.4 *CARMN* inhibits the proliferation, invasion and metastasis of colorectal cancer with mutant *p53*

**FIGURE 4:**
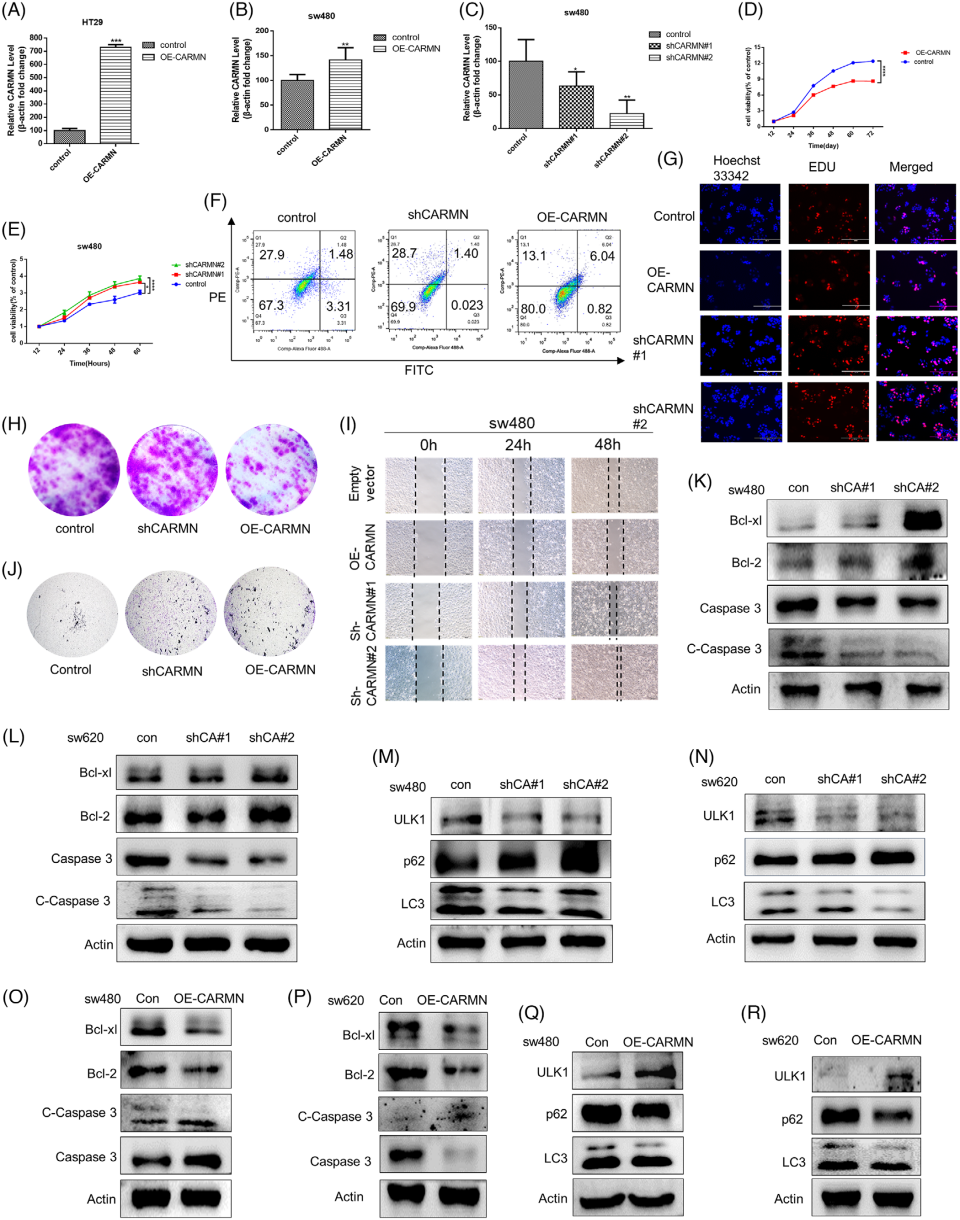
Downregulation of *CARMN* inhibits autophagy and apoptosis, thus promoting the proliferation and migration of CRC cells in vitro. (A–C) RNA expression of *CARMN* in SW480 or HT29 cells transfected with shRNA‐*CARMN* or OE‐*CARMN*. (D, E) CCK8 assays were obtained to measure the effect on SW480 cells transfected with shRNA‐*CARMN* or OE‐*CARMN*. (F) Apoptosis in SW480 cells transfected with sh*CARMN* or OE‐*CARMN* was detected by flow cytometry. Percentages of early apoptosis cells were shown in two right quadrants. (G) EdU assay was obtained to observe the cell proliferation ability in SW480 transfected with OE‐*CARMN* or shRNA‐*CARMN*. (H) Colony formation assays were obtained to measure the effect on SW480 cells transfected with shRNA‐*CARMN* or OE‐*CARMN*. (I, J) Wound‐healing (I) and transwell migration assays (J) were used to observe the cell migration ability in SW480 cells transfected with OE‐*CARMN* or shRNA‐*CARMN* in SW480 cells. (K, L) The apoptosis‐related proteins were detected after the *CARMN* was knockdown in SW480 and SW620 cells. (M, N) Knockdown of *CARMN* made the effect on cell autophagy in SW480 and SW620 cells. (O, P) Expressions of *Bcl‐xl, Bcl‐2, caspase 3* and *c‐caspase 3* following *CARMN* overexpressed in SW480 and SW620 were evaluated by Western blotting. (Q, R) Expressions of *ULK1, P62* and *LC3*II/I following *CARMN* overexpressed in SW480 and SW620 were evaluated by Western blotting.

The lack of *lnc273‐31* or *lnc273‐34* significantly delayed colorectal cancer of mutant *p53R273H* initiation and tumourigenic in vivo.^43^ To clarify the function of *CARMN* in colon cancer cells with mutant *p53*, it was overexpressed by pcDNA3.1 plasmid or knocked down by synthesised specific shRNAs (Figure 4A‐C). As a result, cell viability and colony formation decreased significantly after overexpressing *CARMN*, which increased markedly after knocking down *CARMN* (Figure 4D, E, G and H). Similar results were obtained from scratch assay (Figures 4I and S4E) and the transwell invasion (Figure 4J). To further assess the role of *CARMN* expression induced apoptosis in cells with mutant p53. We evaluated *CARMN* expression in SW480 cells that were either transfected with overexpression of *CARMN* or that were treated with knockdown of *CARMN* in the colon cancer cells with mutant p53. Flow cytometry indicated that increased levels of *CARMN* prevent apoptosis (Figure 4F). The decision to depict only shCARMN#2 was based on its more pronounced knockdown effect compared to shCARMN#1, as evidenced by the construction effect shown in Figure 4E, G, I and K‐N. Subsequent experiments utilised shCARMN#2 to attain more robust results in Figure 4F, H and J. Moreover, *CARMN* inhibited the growth of colorectal cancer cells by inducing S‐phase cell cycle arrest and apoptosis (Figure S4D). These results indicated that *CARMN* inhibited colon cancer cell proliferation and metastasis. Interestingly, *CARMN* had negative feedback on mutant *p53* expression (Figure S4A‐C), which might be through transcriptional regulation. These results above indicated that *CARMN* prevents colorectal cancer with mutant *p53* from proliferation and migration.

The levels of *caspase 3* and *c‐caspase 3* (caspase‐3 p19 subunit to p17 subunit), typical indicators of mitochondria‐mediated apoptosis, were significantly downregulated by knocking down *CARMN*. While the activations of *bcl‐xl* and *bcl‐2* increased, which were detectable in SW480 and SW620 cells (Figure 4K and L). The contrary trends of *caspase 3, c‐caspase 3, bcl‐xl* and *bcl‐2* were observed with overexpressing *CARMN* (Figure 4O and P). In parallel, whether *CARMN* affected autophagy was also studied. As shown in Figure 4M and N, *ULK1* and *LC3II/I* decreased obviously when *CARMN* was knocked down. However, *p62* decreased as the direct downstream target of autophagy. Contrary results were obtained when *CARMN* was overexpressed (Figure 4Q and R). Together, the above results indicated that *CARMN* induced apoptosis and autophagy at the same time.

### 3.5 *MiR‐5683* suppressed the progression of colorectal cancer at downstream of *CARMN*

**FIGURE 5:**
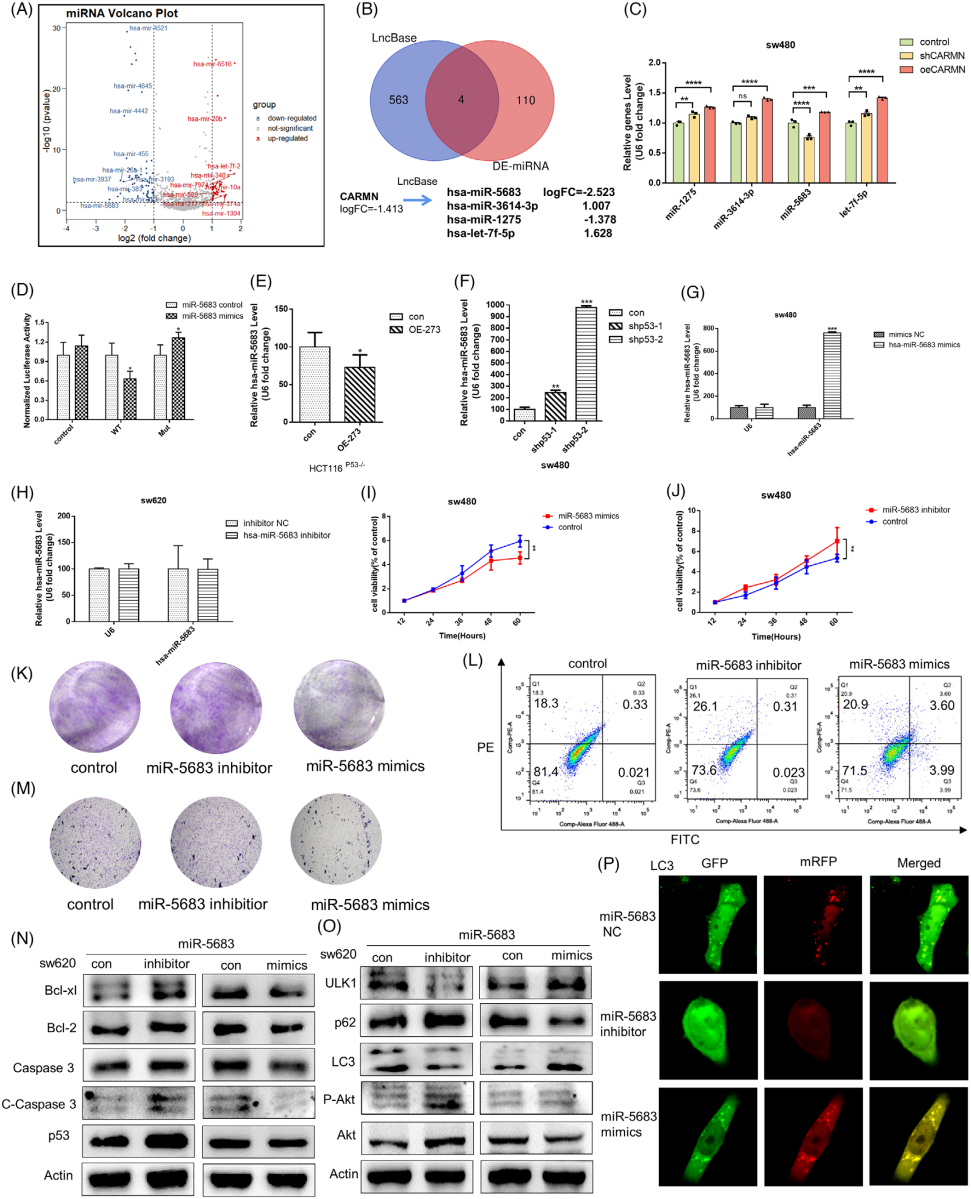
*MiR‐5683* combines with LncRNA *CARMN*, and downregulation of it enhances the proliferation capability and tumour growth of colorectal cancer with mutant *p53*. (A) A volcano plot displayed the DE‐miRNAs between *TP53* mutant and wild‐type patients in colorectal cancer. (B) The Venn diagram was drawn to take an intersection for miRNAs by the bioinformatics tool Venn. (C) The differentially expressed miRNAs identified in (B) were detected in SW480 cells with either knockdown or overexpressed of CARMN. (D) The association between *miR‐5683* and *CARMN* was determined by the luciferase activities in SW480 cells cotransfected with WT‐*CARMN* or MUT‐*CARMN* and *miR‐5683* mimics. (E, F) The expression of *miR‐5683* was measured by RT‐PCR in HCT116^−/−^ cells transfected with OE‐*p53R273H* (E) and SW480 cells transfected with shRNA‐*p53* (F). (G, H) The expression of *miR‐5683* was measured by RT‐PCR in SW480 and SW620 cells transfected with *miR‐5683* mimics (G) or *miR‐5683* inhibitors (H). (I–K, M) The proliferation and migration abilities of SW480 cells transfected with *miR‐5683* mimics or *miR‐5683* inhibitors were tested by CCK8 (I, J), colony formation assays (K), and transwell migration assays (M). (L) Dot plots of Annexin V and PE after transfected with *miR‐5683* mimics and *miR‐5683* inhibitors in SW480 cells. (N, O) The apoptosis‐related and autophagy‐related proteins were detected in SW620 cells by Western blotting. (P) The SW480 cells cotransfected with GFP‐mRFP‐*LC3* and *miR‐5683* mimics or GFP‐mRFP‐*LC3* and *miR‐5683* inhibitors were observed by confocal microscopy.

MiRNAs expression was linked to the gain‐of‐function of mutant *p53* in patients with head and neck squamous cell carcinoma (HNSCC) patients.^44^ Herein, the miRNA expression was screened, showing significant differences as depicted in the volcano plot within the p53 mutational and wild‐type landscape of colorectal cancer samples (Figure 5A). Additionally, we predicted miRNAs that could bind with *CARMN* using the publicly available websites (https://starbase.sysu.edu.cn/starbase2/mirLncRNA.php) and selected the top miRNAs of *miR‐5683* for further analysis (Figure 5B). The examination has uncovered a positive correlation between *miR‐5683* and *CARMN*, with *miR‐5683* decreasing as *CARMN* falls and increasing as *CARMN* rises. Conversely, *miR‐1275* and *let‐7f‐5p* levels increase with decreasing *CARMN*, while they rise with increasing *CARMN* levels. Interestingly, the significant correlation was observed only between *miR‐5683* and *CARMN*, whereas no significant correlation was found between *CARMN* and *miR‐1275*, or *let‐7f‐5p* (Figure 5C). Consistently, mutant *p53R273H* inhibited the expression of *miR‐5683*, whereas *miR‐5683* was obviously upregulated by silencing mutant *p53R273H* in colon cancer cells (Figure 5E and F). Moreover, we discovered that overexpression of *miR‐5683* reduced the luciferase activity of wild‐type *CARMN* but not the Mut‐*CARMN* utilising dual‐luciferase reporter assays (Figures 5D and S5A). It indicated that *CARMN* directly interacted with *miR‐5683*. To investigate the potential role of *miR‐5683* in colorectal cancer cells with mutant *p53*, *miR‐5683* mimics or *miR‐5683* inhibitors was transfected into cells to overexpress or knock down *miR‐5683* (Figures 5G and H and S5D‐F). As a result, cell viability declined with *miR‐5683* overexpression, while it rose after it was knocked down (Figure 5I and J), suggesting that *miR‐5683* suppressed colorectal cancer cell growth. This conclusion was also confirmed by the colony formation (Figure 5K) and EdU immunofluorescence staining (Figure S5G). Quantification of apoptosis in colon cells with mutant *p53* using a standard FACS‐based apoptosis assay that measures the labelling of SW480 cells. As shown in Figure 5L, *miR‐5683* mimics induced colon cells with mutant *p53* apoptosis. However, *miR‐5683* inhibitors showed the opposite effects. Moreover, the flow cytometry experiment confirmed S arrest in the *miR‐5683* inhibition group, while the group of *miR‐5683* mimics resulted in a lower proportion of mutant *p53* cells in the S phase (Figure S5J). In addition, transwell migration assays revealed that *miR‐5683* inhibited colorectal cancer cell metastasis (Figure 5M).

To further determine the function of *miR‐5683* with mutant *p53*, various markers of apoptosis and autophagy were detected. Overexpressed *miR‐5683* extremely decreased the level of *Bcl‐xl* and *Bcl‐2*. Whereas inhibition of *miR‐5683* increased the level of *Bcl‐xl* and *Bcl‐2* and blocked the *c‐caspase 3* induction (Figures 5N and S5H). Interestingly, *miR‐5683* mimics did change with *LC3*II/I induction (Figures 5O and S5I). Furthermore, after transfecting the RFP‐GFP‐*LC3* plasmid into SW480 cells and treating them with *miR‐5683* mimics, GFP degradation, and RFP/GFP elevation were observed, which implied the activation of autophagy (Figure 5P). Interestingly, *miR‐5683* overexpression suppressed mutant *p53R273H* production while inhibition of it clearly boosted mutant *p53R273H* (Figure S5B and C). These results demonstrated that *miR‐5683* had negative feedback on mutant *p53* expression, as *CARMN* did. These suggested that *miR‐5683* inhibits cell progression and contributes to apoptosis and autophagy in colorectal cancer with mutant *p53*.

### 3.6 *MiR‐5683* inhibited colon cancer growth and mutant *p53* activity through degrading *FGF2* mRNA

**FIGURE 6:**
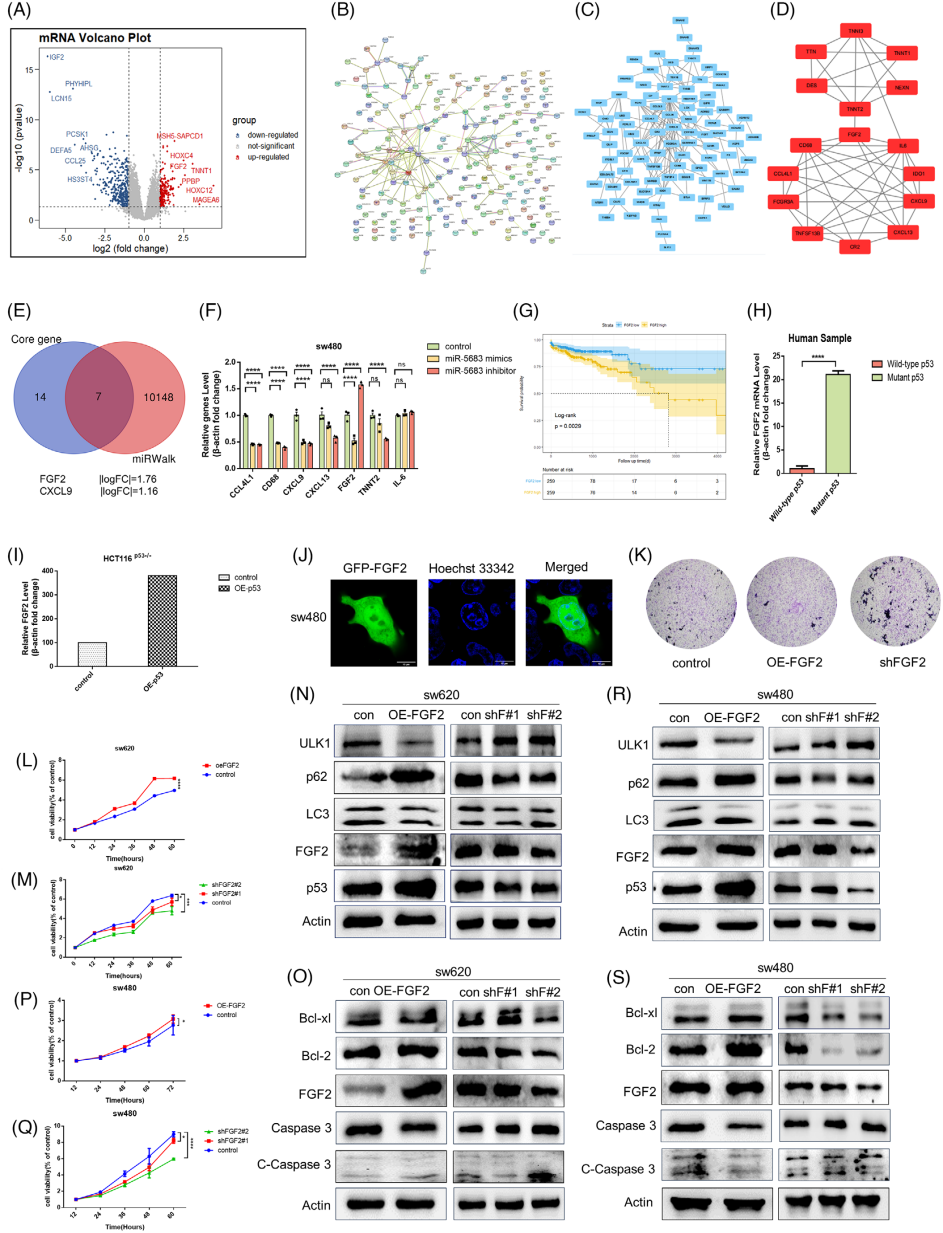
*FGF2* is correlated with *miR‐5683* and has a promotional effect on tumour progression. (A) Upregulation (red) and downregulation (blue) genes were displayed on the volcano plot with mutant *p53* of colorectal cancer. (B) Protein–protein interaction (PPI) networks between the De‐mRNAs were constructed by the online tool STRING. (C) The PPI network was simplified by Cytoscape, which was used to calculate the degree value of De‐mRNAs in PPI networks. (D) The core genes in this PPI network. (E) The Venn plot was used to obtain core genes that combined with *miR‐5683*. (F) The expression levels of these selected genes in (E) were compared in SW480 cells transfected with control mimics (mimics NC), and miR‐5683 mimics, revealing differential expression. (G) The survival plot revealed higher expression of *FGF2* resulted in lower survival. (H) The box diagram displayed the expression of *FGF2* in *TP53* mutant and wild‐type patients. (I) The expression of *FGF2* was measured by RT‐PCR in HCT116^−/−^ cells transfected with OE‐*p53R273H*. (J) The location of *FGF2* was observed by confocal microscopy in SW480 cells transfected with GFP‐*FGF2*. (K–M, P, Q) Transwell migration assays (K) and CCK8 assays (L, M, P, Q) were used to detect the effect on SW480 cells transfected with shRNA‐*FGF2* or OE‐*FGF2*. (N, R) Expression of ULK1, P62, and LC3II/I were obtained by Western blotting in SW480 and SW620 with FGF2 overexpressed or knockdown. (O, S) Overexpression of *FGF2* suppressed autophagy in CRC cells.

Over 2000 miRNAs have been found in humans, and they may regulate one‐third of the mRNAs.^45‐^ ^47^ To find out genes sharing the regulatory role of *miR‐5683* with *CARMN*, we predicted the target genes of *miR‐5683* using the miRWalk database (Figure 6A). Then, the STRING database depicted these significantly different predicted mRNAs with mutant *p53* by protein–protein interaction (PPI) network. The discovery of miRNA‐mRNA target interactions is critical for understanding the regulatory network mediated by *miR‐5683* (Figure 6B and C). Among them, the core genes with a fold change were screened out and investigated by the Cytoscape database (Figures 6D and E and S6A). *FGF2* exhibits an inverse relationship with *miR‐5683*. Specifically, overexpression of *miR‐5683* led to decreased levels of *FGF2*, *CCL4L1*, *CD68* and *CXCL9*. Conversely, inhibition of *miR‐5683* only resulted in a slight increase in FGF2 levels (Figure 6F). Hence, *FGF2* caught our attention because the regulatory network mediated by *miR‐5683* was relatively significant among the predicted target genes. Additionally, Kaplan–Meier survival analysis revealed that patients with high *FGF2* expression had worse survival (Figure 6G).

The function of mutant *p53* increases the activation of surrounding fibroblasts to inhibit autophagy, accompanied with higher *FGF2*.^48^ We compared the *FGF2* levels in TCGA between the *p53* mutant and wild‐type groups and found that the *p53* mutant group of colorectal cancer had higher *FGF2* level, *p* = .0046 (Figures 6H and S6B). Furthermore, the relationship between mutant *p53* and *FGF2* was studied. The result showed that overexpressed mutant *p53R273H* evidently upregulated the expression of *FGF2* (Figure 6I).

To investigate the mechanism by which *FGF2* exerts its function, the subcellular localisation of *FGF2* was initially detected. GFP‐FGF2 was transfected into sw480 cells and visualised FGF2 localisation using confocal microscopy (Figure 6J). Moreover, the subcellular localisation of FGF2 was confirmed by an immunofluorescence assay using FITC‐labelled immunostaining with FGF2 antibodies. These findings revealed that FGF2 localises within both the nucleus and cytoplasm, as depicted in Figure S6E. Next, the colony formation assay, transwell assay and CCK‐8 experiments indicated that *FGF2* promoted colorectal cancer cell proliferation (Figures S6C and 6K–M, P and Q). *FGF2* could protect cells against other kinds of death such as apoptosis or necrosis through autophagy suppression.^49^ To further explore the mechanism behind, apoptosis and autophagy were analysed. The results showed that overexpressing *FGF2* distinctly increased *Bcl‐xl*/*Bcl‐2*/*p62* expression, while obviously decreased the expression of *caspase 3*/*ULK1*/ *LC3*II/I (Figure 6N, O, R and S). Contrary trends were observed when *FGF2* was knocked down (Figure 6N, O, R and S), which demonstrated that *FGF2* inhibited both apoptosis and autophagy. To confirm that *miR‐5683* might exert its biological function by *FGF2*, a direct interaction of *miR‐5683* and *FGF2* was further substantiated (Figure S6D). These findings indicated that *miR‐5683* downregulates *FGF2* and activates apoptosis and autophagy.

### 3.7 *CARMN* collaborates with *miR‐5683* to downregulate *FGF2* and induce autophagy

**FIGURE 7:**
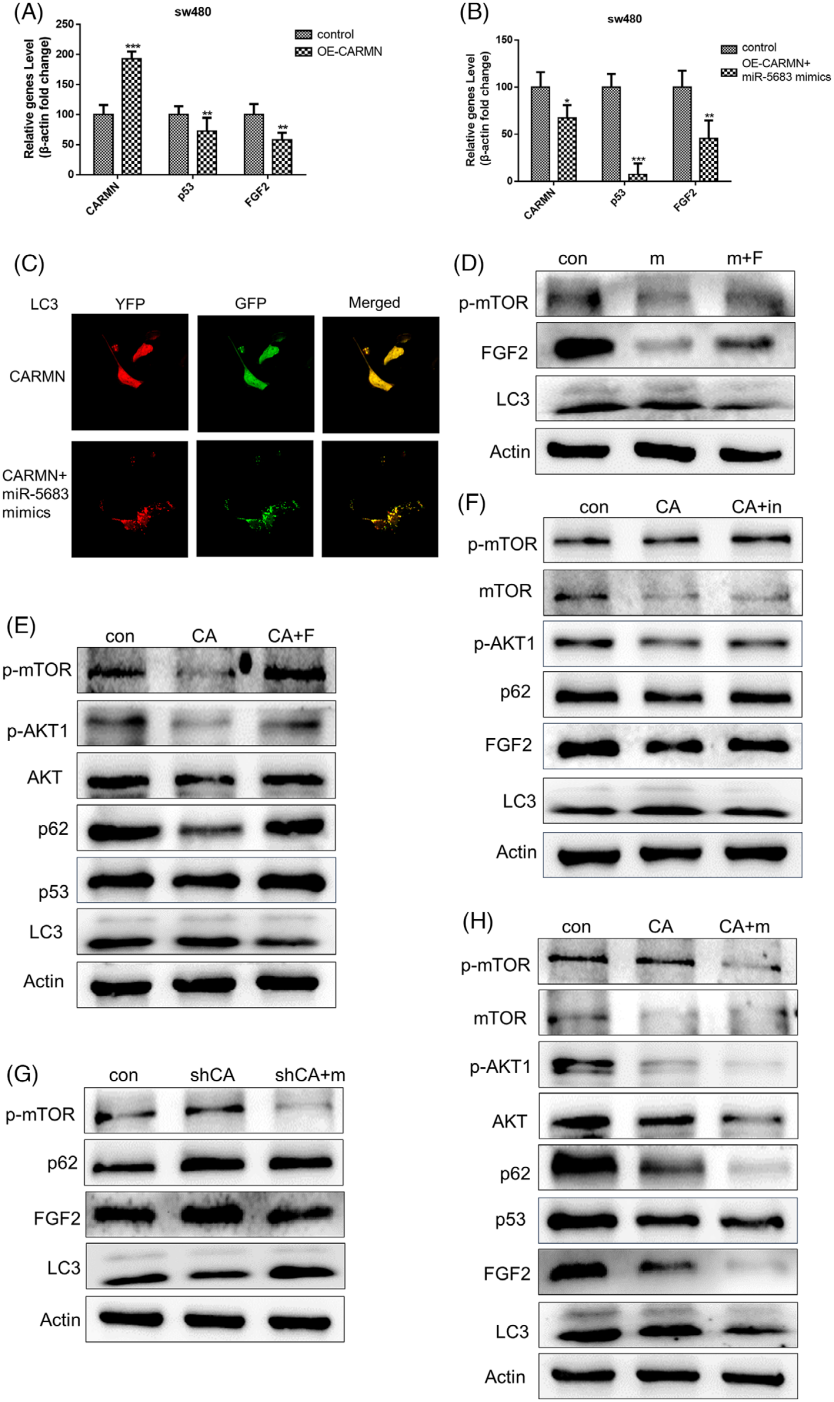
*CARMN* and *miR‐5683* combine to downregulate the expression of *FGF2*. (A) RT‐PCR assays were used to calculate the expression of *FGF2, p53* and *CARMN* in SW480 cells transfected with OE‐*CARMN*. (B) *FGF2* and *p53* expression levels were reduced effectively in SW480 cells cotransfected with OE‐*CARMN* and *miR‐5683* mimics. (C) The cells were observed by confocal microscopy in SW480 cotransfected with YFP‐*LC3* and GFP‐*CARMN* or YFP‐*LC3*, GFP‐*CARMN*, and *miR‐5683* mimics. (D–H) *CARMN* (E, F, H) and *miR‐5683* (D, G, H) overexpression in SW480 cells reduced phosphorylation of mTOR and AKT1, thereby triggering the downregulation of FGF2 and p53, consequently enhancing cell autophagy. Notably, inhibition of *CARMN* (G) resulted in decreased LC3II/I and increased expression of p‐mTOR, p62, and FGF2 expression. Furthermore, it was observed that the expression levels of p‐mTOR, p‐Akt and FGF2 were elevated upon overexpression of *FGF2* (D, E).

Our results have demonstrated that *miR‐5683* downregulating *FGF2* expression on both protein and mRNA levels (Figures S5H and I and S7A and B), which verified that *FGF2* was the target gene of *miR‐5683*. Interestingly, as the upstream regulator of *miR‐5683*, *CARMN* overexpressing also reduced *FGF2* expression (Figures 7A and S7C). According to Zhao et al., the role of multiple autophagic pathways were provided in targeting and degrading mutant p53 proteins.^50^ MiRNAs generated from mutant p53 play a critical role in autophagy inhibition.^51^ To further reveal their relation and function, *CARMN* and *miR‐5683* were co‐overexpressed, which led to a further reduction of *FGF2* and mutant *p53* expression on both mRNA and protein level, compared with that of the *CARMN* overexpressing only group (Figure 7A and H). Meantime, *p62* also had a further reduction while *LC3*II/I had a further increase, and more YFP‐*LC3* puncta was observed under the same condition (Figure 7H and C). These results indicated that *CARMN* and *miR‐5683* have a synergistic effect on *FGF2* inhibition, autophagy induction and negative feedback of mutant *p53*.

*FGF2* has been reported to promote tumourigenesis via stimulating the *PI3K/Akt* signalling pathway.^43^, ^50^, ^51^ And *mTOR* is the critical downstream effector of the *Akt* pathway and upstream of p70 S6 kinase.^52^ Thus, it was selected for further analysis. To clarify the function of *CARMN* and *miR‐5683*, the overexpression and interference efficiency of *CARMN*, *p53* and *FGF2* were assessed by qPCR analysis. The assay showed that overexpression of *CARMN* and *miR‐5683* in SW480 cells dramatically decreased *p53* expression compared with the overexpressed *CARMN* group. Interestingly, overexpression of *CARMN* and *miR‐5683* could inhibit *FGF2* expression than that in the overexpressed *CARMN* group (Figure 7A and B). After overexpression of *CARMN*, *miR‐5683*, and treatment with YFP‐*LC3*, we detected more distribution of *LC3* in living cells, suggesting the autophagy synergistic effect of *CARMN* and *miR‐5683* (Figure 7C).

As a result, we investigated the ability of modulation of the *CARMN*‐*miR‐5683*‐*FGF2* axis to influence *Akt* pathway activation. To investigate the prospective autophagic strategies of targeting mutant *p53* in cancer, we transfected SW480 cells with *CARMN*, *miR‐5683*, and *FGF2*. The results showed that *CARMN* and *miR‐5683* were related to the *Akt/mTOR* signalling pathway, which plays a pivotal role in the oncogenesis of colorectal cancer cells mutant *p53*. Our result revealed that phosphorylated Akt (*P‐Akt1*) decreased in *CARMN*‐transfected cells compared with control cells. To investigate the effect of *CARMN* and *miR‐5683* on the *mTOR* pathway, phosphorylated mTOR (*P‐mTOR*) was detected. *P‐mTOR* was reduced in *CARMN*‐transfected cells compared with vectors. Conversely, *P‐mTOR* was increased in inhibition of *CARMN* cells than vectors. The promotion effect of *CARMN* and *miR‐5683* on the *Akt/mTOR* pathway was abolished in *miR‐5683* inhibited or *FGF2* overexpressed cells (Figure 7D‐H). These findings strongly suggested that *CARMN* and *miR‐5683* could promote the *Akt/mTOR* pathway.

### 3.8 *CARMN* suppressed colon cancer growth in vivo and could be used as a potential tumour inhibitor

**FIGURE 8:**
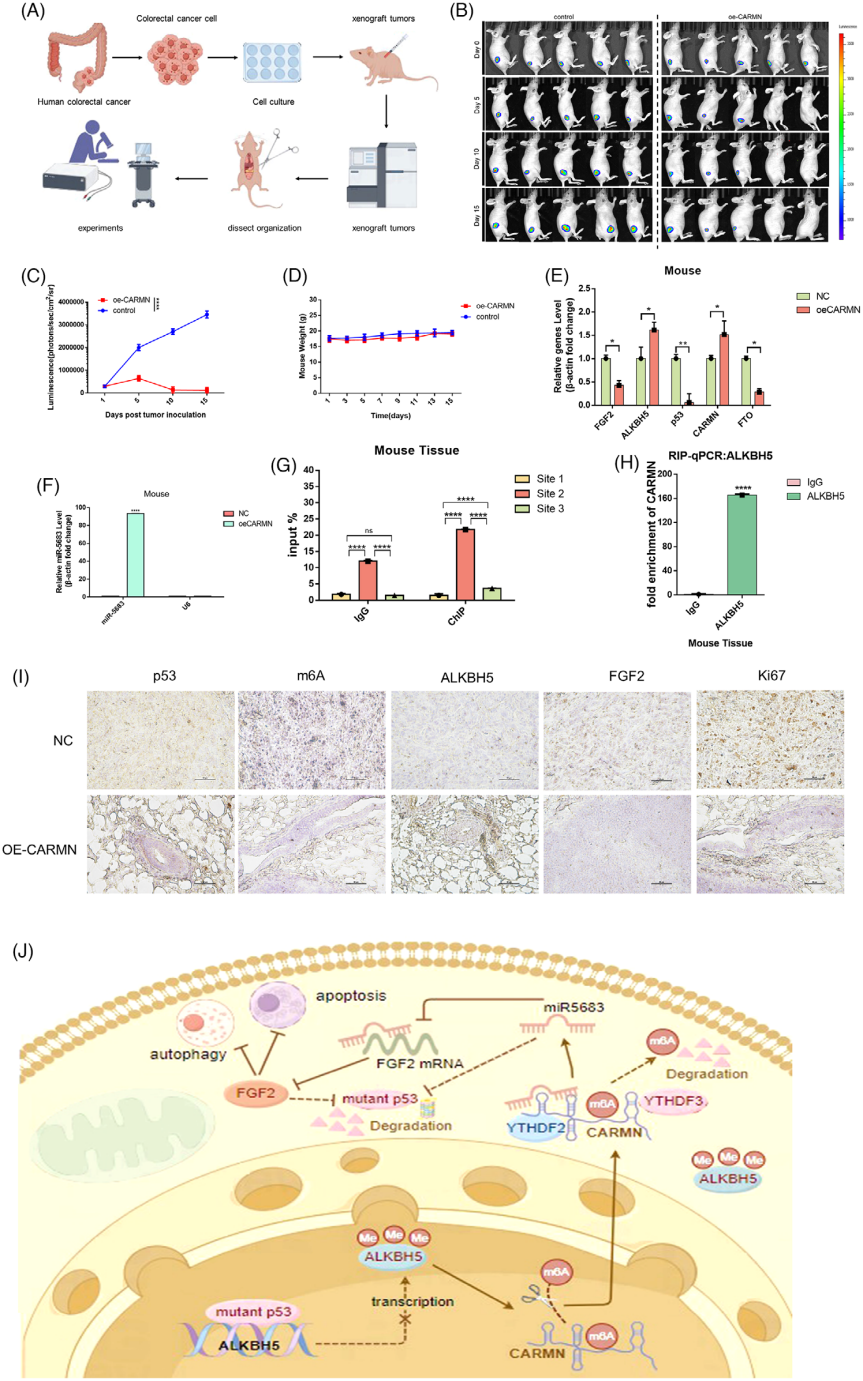
*CARMN* mediates in vivo antitumour effects. (A–D) Nude mice were used to establish a xenograft model with control or OE‐*CARMN* tumour. (B–D) tumours pictures in this test, (B, C) tumour volume or (D) mouse weight was measured. *p < .05. (E, F) RT‐PCR analysis of the expression levels of *CARMN*, *FGF2, ALKBH5, p53, FTO* and *miR‐5683* in the implanted tumours. (G) ChIP‐qPCR analysis conducted on mouse tissue tested three sites of mutant p53 binding to the ALKBH5 promoter, with IgG serving as an internal negative control. (H) The correlation between *CARMN* and *ALKBH5* using RIP assay with *ALKBH5* antibody in mouse xenograft tumour tissue containing mutant p53. (I) Representative data from immunohistochemistry staining of *p53, m6A, ALKBH5, FGF2* and *Ki67* in subcutaneous transplantation tumours obtained from nude mice after injection with Luc‐*CARMN* and Luc‐control SW480 cells. (J) Schematic representation of *ALKBH5*‐mediated *CARMN* induction to regulate *miR‐5683/FGF2* signalling pathway.

To verify the effect of *CARMN* on colorectal cancer growth in vivo, vector, and *CARMN* green fluorescent protein (GFP) were knock‐in (KI) in SW480 cells, which were then subcutaneously inoculated in the right armpit of the nude mouse to construct xenografts tumour models, respectively (Figure 8A). Significantly lower tumour volume was detected in SW480/overexpressing *CARMN* group compared to that of SW480/vector group (Figure 8B and C), while the weight of these mice in the two groups had almost no difference (Figure 8D). Furthermore, qRT‐PCR analysis revealed the expression levels of p53, CARMN, ALKBH5, FTO and FGF2 in mouse tissues. The expression of FGF2, mutant p53 and FTO was diminished in the mouse xenograft group overexpressing CARMN compared with the control group (Figure 8E). The result indicated that *CARMN* suppressed colorectal cancer growth in vivo, in the presence of mutant *p53*. Specifically, the group of overexpressed *CARMN* has higher levels of *miR‐5683* than control in vivo (Figure 8F). We also assessed the levels of *p53, m6A, ALKBH5, FGF2* and *Ki67* and their correlations in subcutaneous transplanted tumours in mice. Tumours overexpressing *CARMN* showed a higher level of *ALKBH5* and a lower level of *p53, m6A, FGF2* and *Ki67* than control tumours (Figure 8I). Collectively, our results demonstrated that mutant *p53* transcriptionally downregulated *ALKBH5* expression, which led to higher‐level m6A methylation of *CARMN*, subsequently degraded by *YTHDF2/3*. On the other hand, *CARMN* directly interacted with *miR‐5683* and they had a synergistic effect on colorectal cancer growth suppression, through degrading *FGF2* mRNA to inhibit Akt/mTOR pathway and induce apoptosis/autophagy. Additionally, both *CARMN* and *miR‐5683* had a negative feedback while *FGF2* had a positive feedback on mutant *p53* expression (Figure 8J).

## 4 DISCUSSION

*TP53* mutations gains‐of‐function properties are associated with tumour development and reduced patient survival rates. Our findings demonstrated that mutant *p53* could bind the promoter of *PHLPPL* to inhibit its expression. In addition, colorectal cancer patients with mutant *p53* had high levels of *AKT* phosphorylation and *PD‐L1* expression.^37^ Apoptosis induced by traditional *p53*‐dependent drugs was eliminated in mutant *p53* colon cancer cells, accompanied with increased cell viability. It showed that mutant *p53* could inactivate *PUMA* transcription to promote tumour chemoresistance.^53^ In this study, we identified *ALKBH5* as a posttranscription target of mutant *p53* and a regulator of *CARMN* deacetylation. Moreover, we elucidated the role of the *CARMN/miR‐5683/FGF2* axis in apoptosis and autophagy both in vitro and in vivo for the first time. Mechanically, mutant *p53* could bind to the *ALKBH5* promoter, suppressing its transcription and consequently increasing m6A modification of *CARMN*, leading to its degradation. *CARMN* correlated with *miR‐5683* to downregulate *FGF2* expression, thereby initiating autophagy. Our results highlight the crucial role of *ALKBH5* activation and *CARMN* induction as a promising therapeutic target for colorectal cancer with mutant *p53*.

Mutant *p53* loses its ability to suppress tumour development and gain‐of‐function activities, instead accelerating tumour growth. A poor clinical prognosis is associated with *TP53* mutation, which is present in approximately half of all colorectal cancer cells. Mutant *p53* significantly contributes to 5FU resistance in colorectal cancer.^53^ Currently, lncRNAs have emerged as important regulators of tumour development and progression, influencing apoptosis, DNA damage response, as well as cell proliferation and invasion.^54^ The findings suggest that *MALAT1* plays an important role in regulating *VEGFA* isoform production in breast cancer cells harbouring gain‐of‐function mutant *p53* and ID4 proteins.^55^ However, the potential role of lncRNAs in colorectal cancer with mutant *p53* remains poorly understood. In this study, 222 samples with mutant *p53* and 251 samples with wild‐type *p53* from colorectal cancer patients were analysed. Most samples with mutant *p53* exhibited low levels of *CARMN* (Figure 1A), accompanied by an extremely low level of *ALKBH5* (Figure 1H, I, K and L). Meantime, in contrast to the upregulated *CARMN* expression, patients with lower *CARMN* levels had shorter overall survival in later‐stage colorectal cancer with mutant *p53* and wild‐type *p53* (Figure 1B). Therefore, *CARMN* may act as a tumour suppressor in colorectal cancer with mutant *p53*.

There is also an opinion suggesting that blocking *p53* mRNA m6A modification by S‐adenosyl homocysteine or siRNA‐mediated *METTL3* inhibition increases susceptibility of hepatocellular carcinoma to chemotherapy.^56^ According to Uddin et al., m6A modification at the *p53* pre‐mRNA leads to p53R273H mutant protein expression. Suppressing of RNA methylation and ceramide glycosylation may represent an effective therapeutic strategy for targeting *TP53* missense mutations.^57^ Additionally, *ALKBH5* levels were found to be elevated in the wild‐type p53 group, while the *p53* mutation group exhibited low levels of *ALKBH5* in pancreatic cancer, as revealed by the analysis of TCGA datasets. Furthermore, it was identified that wild‐type *p53* could bind to the *ALKBH5* promoter to activate *ALKBH5* transcription.^27^ Although there have been few studies on m6A modification with mutant *p53* in cancer therapy in recent years, it is conceivable that m6A modification of lncRNAs occurs with mutant *p53* in colorectal cancer. Our results showed that mutant *p53* blocked *ALKBH5* promoter activity, three *p53* binding sites were predicated on the *ALKBH5* promoter, and ChIP assay results revealed that one of these binding sites directly interacted with mutant *p53*. This mutant *p53* binding site differs from the wild type *p53* binding site reported by Guo et al.^27^ The results further confirmed the interaction between mutant *p53* and the promoter of *ALKBH5* (Figures 2C‐L and S2D‐F). These finding likely contribute to understanding the regulatory mechanisms involving mutant p53 and its impact on ALKBH5 expression. In this study, we focus on the demethylase *ALKBH5* regulating the methylation of *CARMN* in colorectal cancer with mutant p53. In contrast to the antisense *CARMN* group, biotin‐labelled *CARMN* obviously pulled down *ALKBH5* (Figure 3F). In addition, a significant upregulation of *CARMN* was observed following *ALKBH5* overexpression (Figure 3K), suggesting that *ALKBH5* plays a critical role in removing the methylation of *CARMN*.

Cell migration and dedifferentiation may be triggered by *CARMN*‐mediated regulation of miRNAs. However, *CARMN* could influence human coronary arterial smooth muscle cells (hCASMCs) proliferation independently of *miR‐143* and *miR‐145*.^15^ *MiR143HG* (*CARMN*) was found to suppress *miR‐1275* levels, which directly targeted *AXIN2* to modulate the Wnt/catenin pathway.^58^ Investigation into the mechanism of *CARMN* in regulating colorectal cancer with mutant *p53* revealed its cooperation with *miR‐5683* to exert its function (Figure 7B, C and H). The role of *miR‐5683* in suppressing gastric cancer by targeting the gene pyruvate dehydrogenase kinase 4 (*PDK4*) has been confirmed.^59^ In this study, it was confirmed that *miR‐5683* downregulated the expression of the wild type *CARMN* group, while this effect was nullified by the mutant sequence of *CARMN* in dual‐luciferase reporter assays (Figure 5D). Moreover, to assess their effect, the expression of *FGF2*, a target of *miR‐5683*, was examined (Figure 6). Various regulators might be involved in *FGF2* expression in different cancers. *Circ001422* and *miR‐195‐5p* have been shown to increase *FGF2* expression to accelerate osteosarcoma tumourigenesis and metastasis.^60^ However, in this study, *FGF2* was significantly downregulated by the synergistic effect of *CARMN* and *miR‐5683* (Figure 7A and B), possibly through the *Akt/mTOR* pathway (Figure 7D‐H).

## 5 CONCLUSIONS

*CARMN* loss is associated with poor clinicopathological characteristics and prognosis of colorectal cancer with mutant *p53*. Overexpression of *CARMN* reduces cell proliferation, migration and colorectal cancer with mutant *p53*, whereas *CARMN* knockdown facilitates mutant *p53* with colorectal cancer progression. Demethylation of *CARMN* and increase in its level underlie the effect of *ALKBH5* in an m6A‐*YTHDF2/YTHDF3*‐dependent manner.

## AUTHOR CONTRIBUTIONS

Nannan Liu and Xinxiu Jiang designed experimental approaches, performed experiments, analysed data and cowrote the manuscript; Ge Zhang, Shuaiyu Long, Jiehan Li, Meimei Jiang, Guiyun Jia and Renyuan Sun performed experiments; Lingling Zhang and Yingjie Zhang analysed data, provided oversight and critical expertise and cowrote the manuscript. All authors read and approved the final manuscript.

## CONFLICT OF INTEREST STATEMENT

The authors declare no conflict of interest.

## ETHICS STATEMENT

The use of clinical specimens and animal experiments were ethically approved by the Clinical Research and Laboratory Animal Ethics Committee.

## Supporting information

Supporting Information

## Data Availability

Data will be made available on request.
