# Supplementary material for: LncRNA CARMN m6A demethylation by ALKBH5 inhibits mutant p53‐driven tumour progression through miR‐5683/FGF2
Source: Clin Transl Med. 2024 Jul 22;14(7):e1777. doi: 10.1002/ctm2.1777 (PMC11263751; doi:10.1002/ctm2.1777)
Supplement: Supplementary file 1 — Supporting Information [file CTM2-14-e1777-s001.docx]

**Supplementary Information for**

**LncRNA *CARMN* m6A Demethylation by *ALKBH5* Inhibits Mutant *p53*-Driven Tumor Progression through *miR-5683/FGF2***

Nannan Liu^1,6^ | Xinxiu Jiang^1,6^ | Ge Zhang^2,3^ | Shuaiyu Long^5^ | Jiehan Li^3^ | Meimei Jiang^1^ | Guiyun Jia^1^ | Renyuan Sun^3^ | Lingling Zhang^2^ | Yingjie Zhang^1,4^*

^1^School of Biomedical Sciences, Hunan University, Changsha, China

^2^Department of Laboratory Medicine, the Third Xiangya Hospital, Central South University, Changsha, China

^3^Department of Gastroenterology, the First Affiliated Hospital of Zhengzhou University, Zhengzhou, 450052, China

^4^Department of Gastroenterology, Huadong Hospital, Shanghai Medical College, Fudan University, Shanghai, 200040, P.R. China

^5^Hebei Provincial Mental Health Center, Hebei Key Laboratory of Major Mental and Behavioral Disorders, The Sixth Clinical Medical College of Hebei University, Baoding, Hebei, 071000, China

^6^These authors contributed equally to this work

***Corresponding authors:**

Yingjie Zhang, Ph.D., email: yingjiezhang@hnu.edu.cn

**This PDF file includes:**

Figs. S1 to S7

**
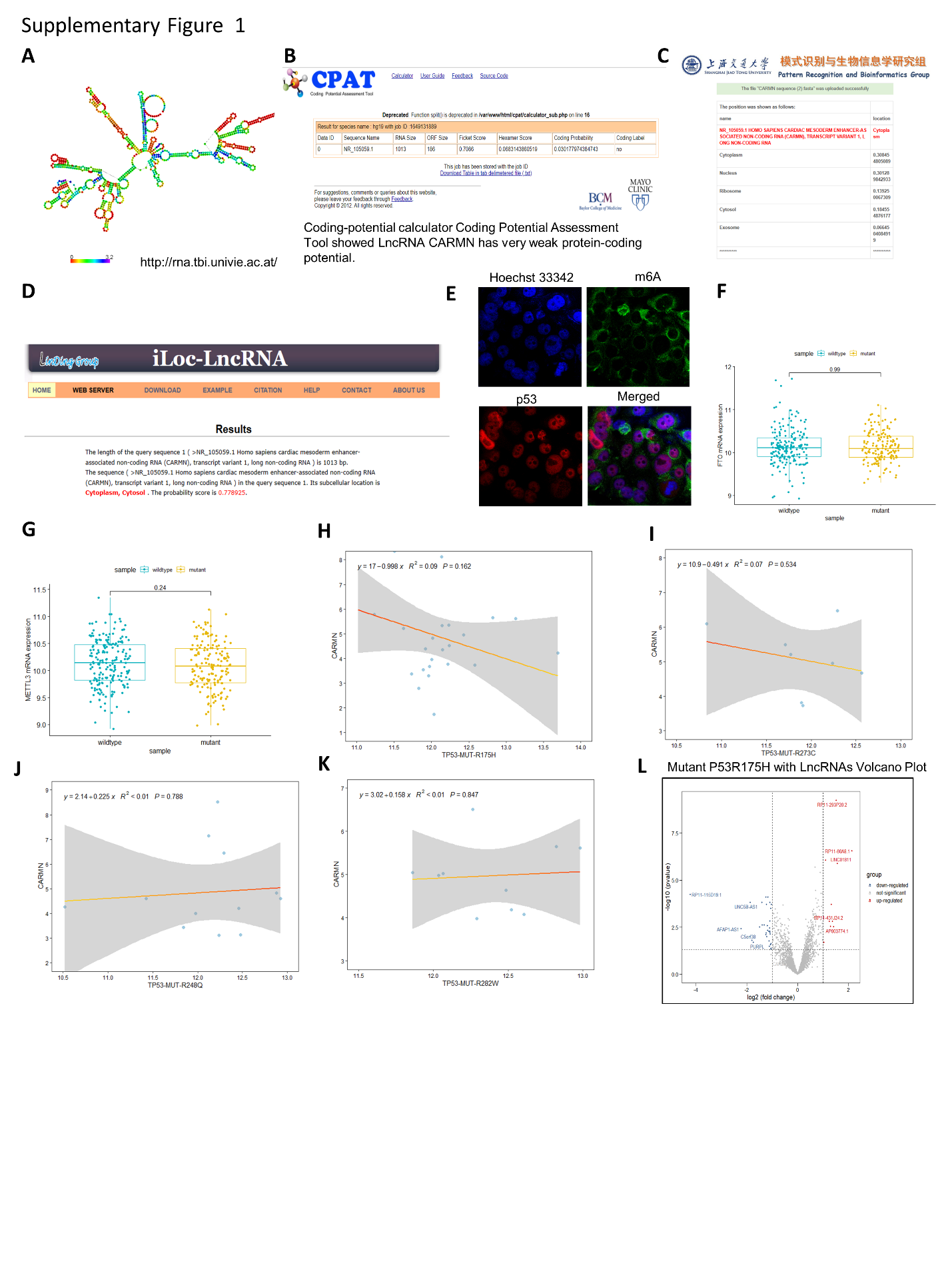
**

**
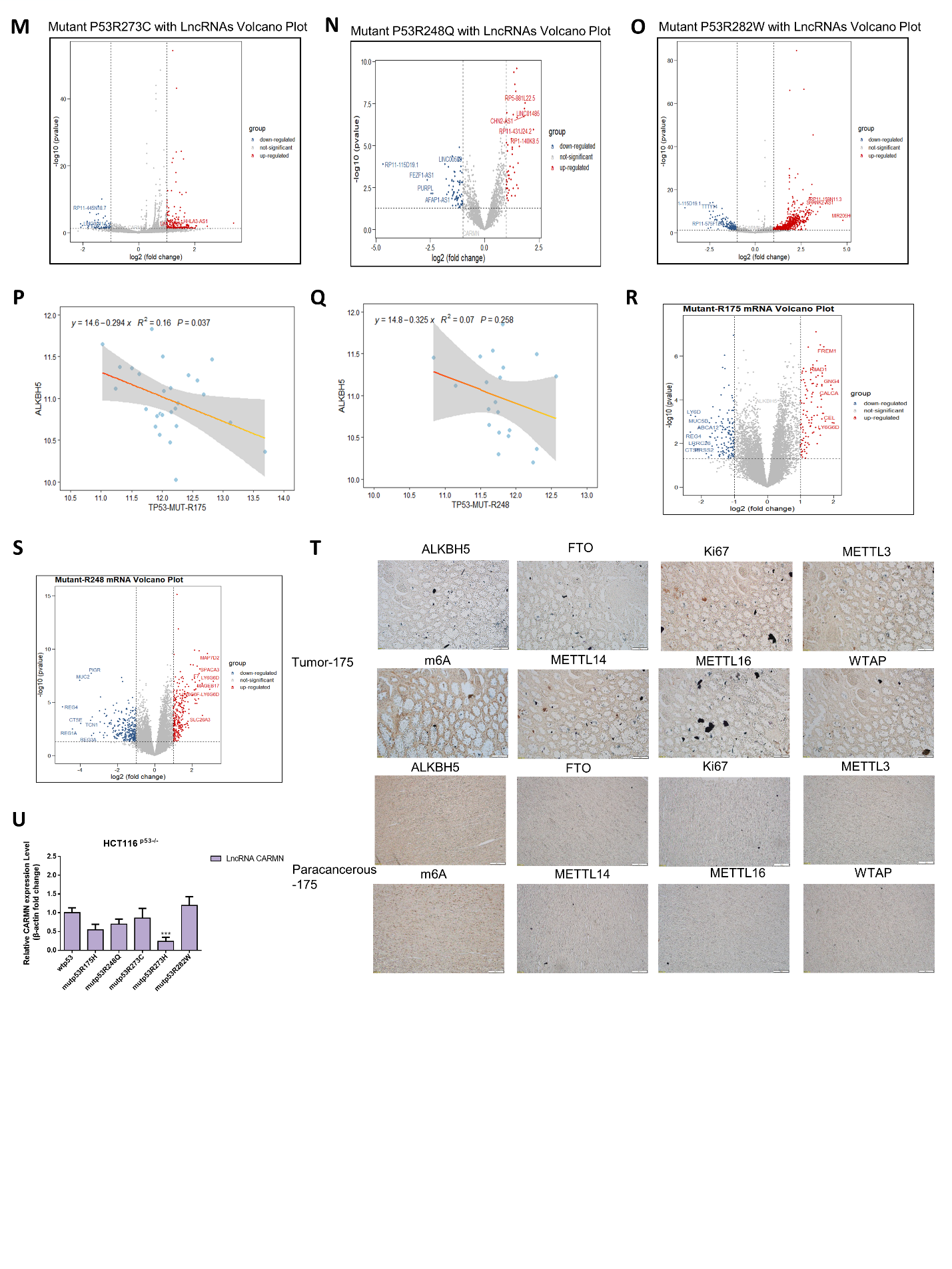
Figure S1.**

(A) The secondary structure of LncRNA *CARMN* was predicted by the online tool (http://rna.tbi.univie.ac.at/). (B) LncRNA *CARMN* was a non-coding RNA in CPAT online tool. (C-D) The location of LncRNA *CARMN* was predicted by the lncLocator (http://www.csbio.sjtu.edu.cn/bioinf/lncLocator/) and iLoc-LncRNA database (http://lin-group.cn/server/iLoc-LncRNA/pre.php). (E) Immunofluorescent analysis of m6A (green) accumulation in SW480 cells with *p53* antibody (red). (F, G) The box plots made in R revealed the expression of *FTO* and *METTL3* without significant differences between mutants and wild-types. (H-O) The correlation between LncRNA CARMN and mutant p53R175H (27 mutant) (H, L), p53R273C (8 mutant) (I, M), p53R248Q (22 mutant) (J, N), p53R282W (11 mutant) (K, O) was assessed and graphically represented employing the lllumina package, utilizing colon cancer data sourced from the TCGA database. (P-S) The correlation between ALKBH5 and mutant p53R175H, p53R248Q was analyzed by corrplot (P, Q) and lllumina (R, S) package with colon cancer data retrieved from the TCGA database. (T) A comparison of *ALKBH5, FTO, METTL3, METTL14, METTL16, WTAP*, and *Ki67* expression levels between tissues with mutant p53 colorectal cancer and adjacent normal tissues.

**
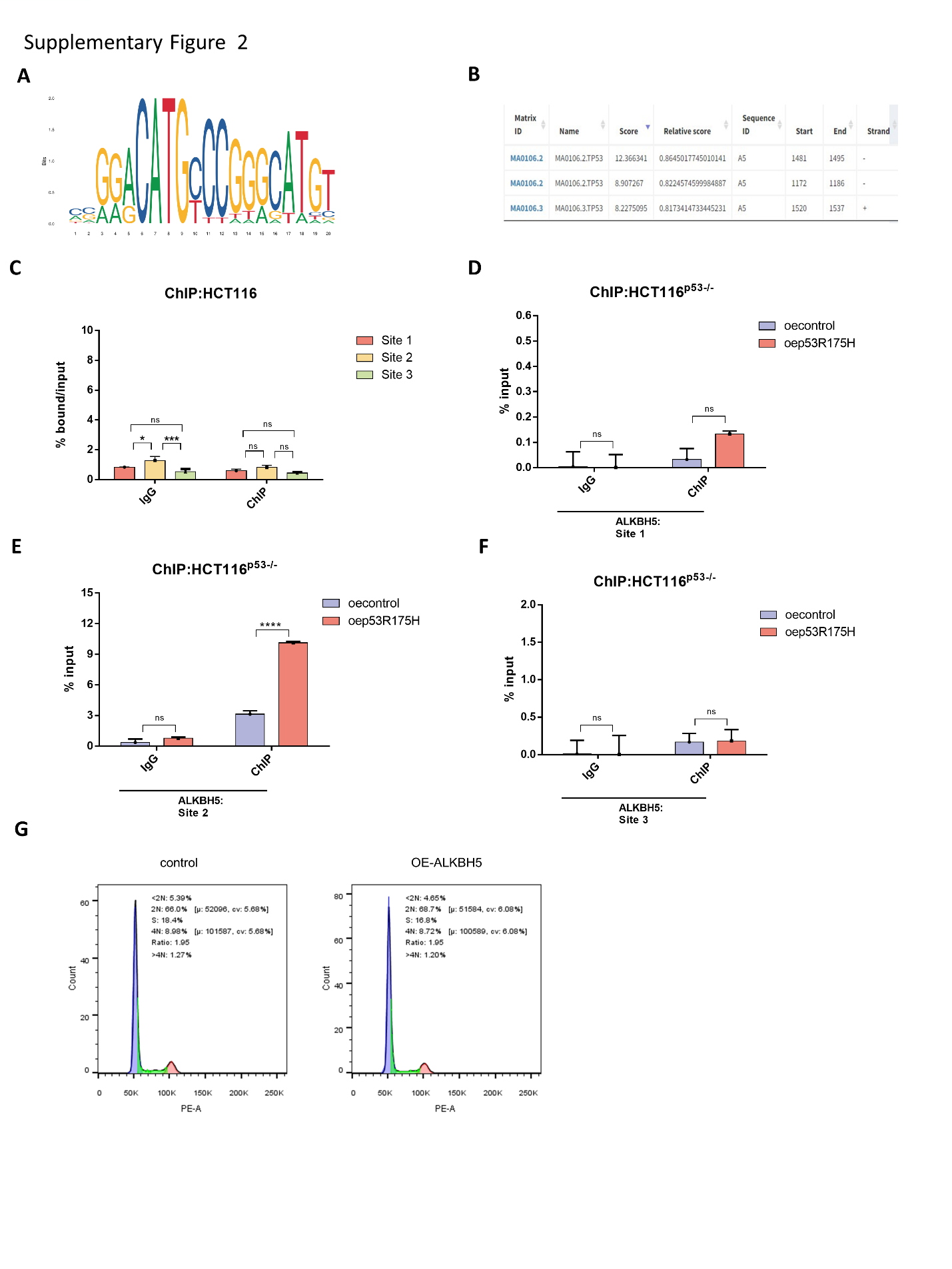
Figure S2.**

(A-B) Motif analysis was made by the online tool JASPAR to identify the *ALKBH5* binding motif of mutant *p53*. (C-F) ChIP-qPCR analysis was performed on HCT116 (C) and HCT116 p53^-/-^ (D-F) cells, confirming the binding of mutant p53 to three sites on the ALKBH5 promoter, while IgG was utilized as an internal negative control. (G) Cell cycle distribution was obtained by flow cytometry in SW480 cells overexpressed *ALKBH5*.

**
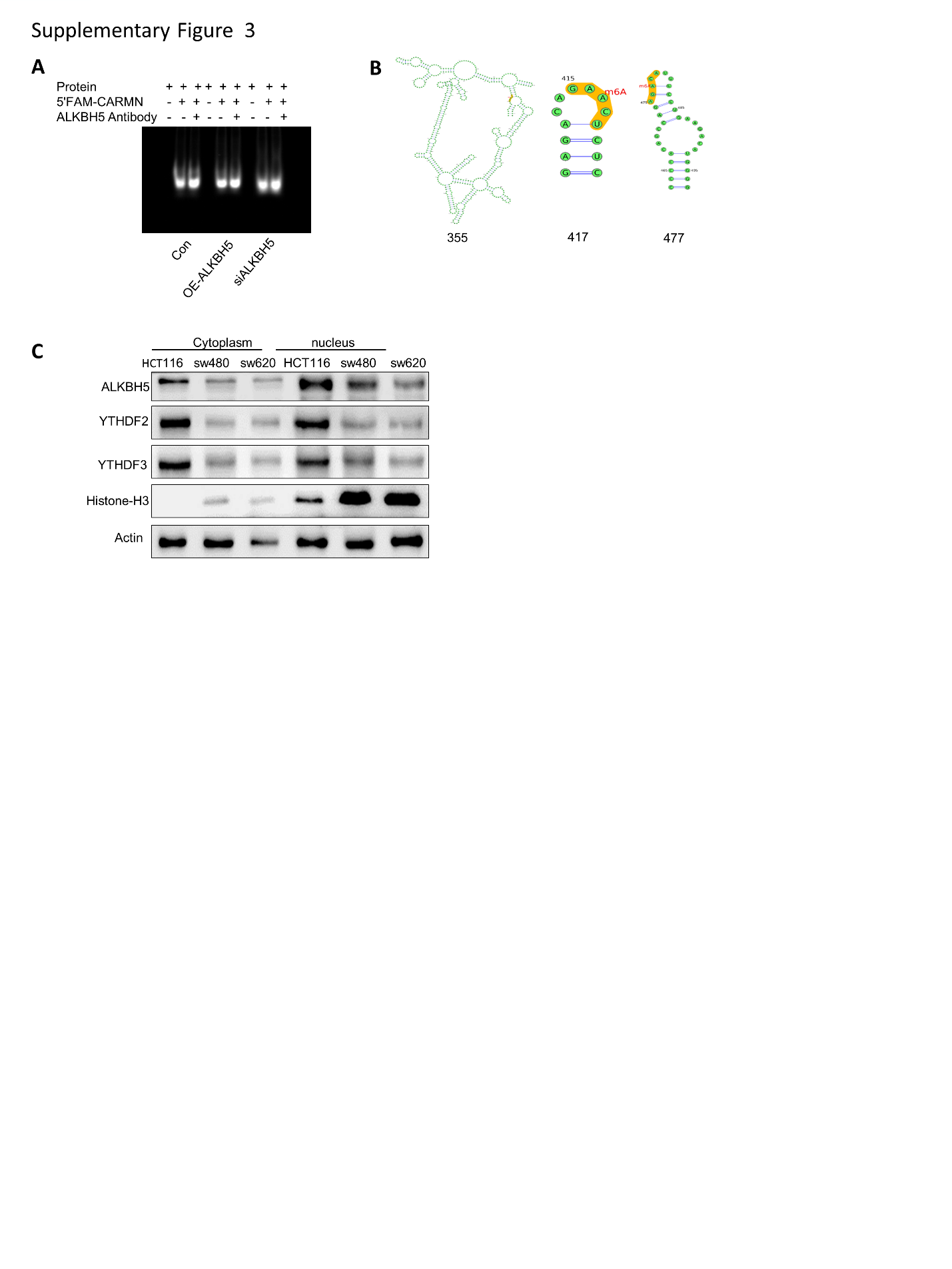
**

**Figure S3.**

(A) Proteins obtained from the RNA pull-down experiment were used to calculate the quantification of *ALKBK5* by EMSA in SW480 cells transfected with OE-*ALKBH5* or si-*ALKBH5*. (B) The secondary structure of m6A sites of CARMN was predicted by the online tool SRAMP (<https://www.cuilab.cn/sramp>). (C) *ALKBH5*, *YTHDF2*, and *YTHDF3* were located by western blotting using the protein extracted from nucleocytoplasmic separation experiment in HCT116, SW480, and SW620 cells.

**
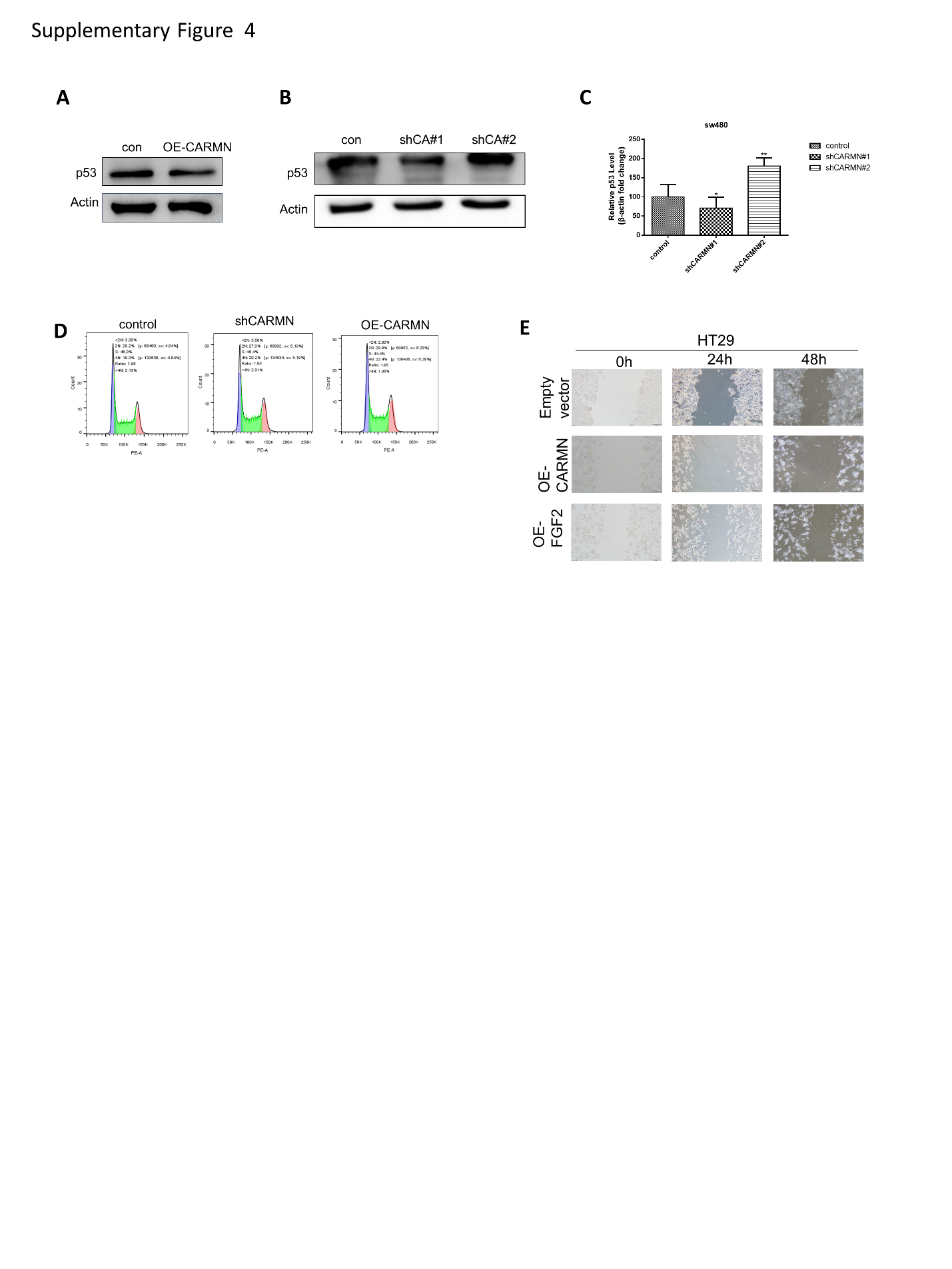
**

**Figure S4.**

(A-B) Expression of p53 was tested by western blotting in SW480 cells transfected with shRNA-*CARMN* or OE-*CARMN*. (C) RT-PCR assays were used to measure the expression of subcellular *CARMN* in the nucleus and cytoplasm of SW480 cells. *β-actin* and *U6* were worked as endogenous controls. (D) Cell cycle distribution was obtained by flow cytometry in SW480 transfected with shRNA-*CARMN* or OE-*CARMN*. (E) Wound-healing assays were used to observe the cell migration ability in SW480 cells transfected with OE-*CARMN* or sh-*CARMN* in HT29 cells.

**
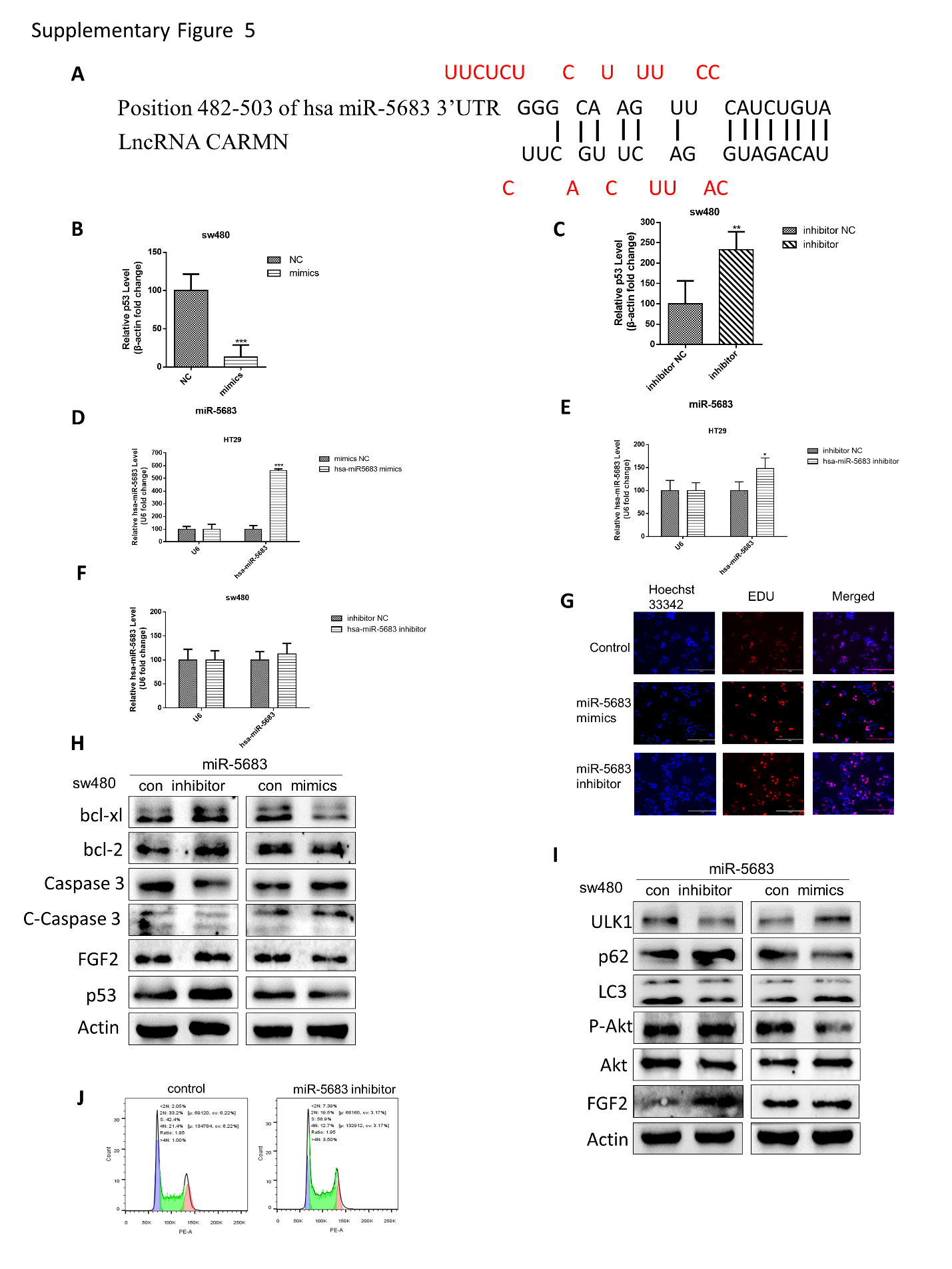
**

**Figure S5.**

(A) The predicted *miR-5683* binding sites in the *CARMN* transcript. (B, C) RT-PCR assays revealed the change of *p53* in SW480 cells transfected with *miR-5683* mimics or *miR-5683* inhibitors. (D-F) Expression of *miR-5683* was obtained by RT-PCR in HT29 or SW480 transfected with *miR-5683* mimics or *miR-5683* inhibitors. (G) EdU assays were applied to compare the cell proliferation ability in SW480 transfected with *miR-5683* mimics or *miR-5683* inhibitors. (H, I) It was used to identify the apoptosis and autophagy related proteins in SW480 cells. (J) Cell cycle distribution was obtained by flow cytometry in SW480 cells transfected with *miR-5683* mimics or *miR-5683* inhibitors.

**
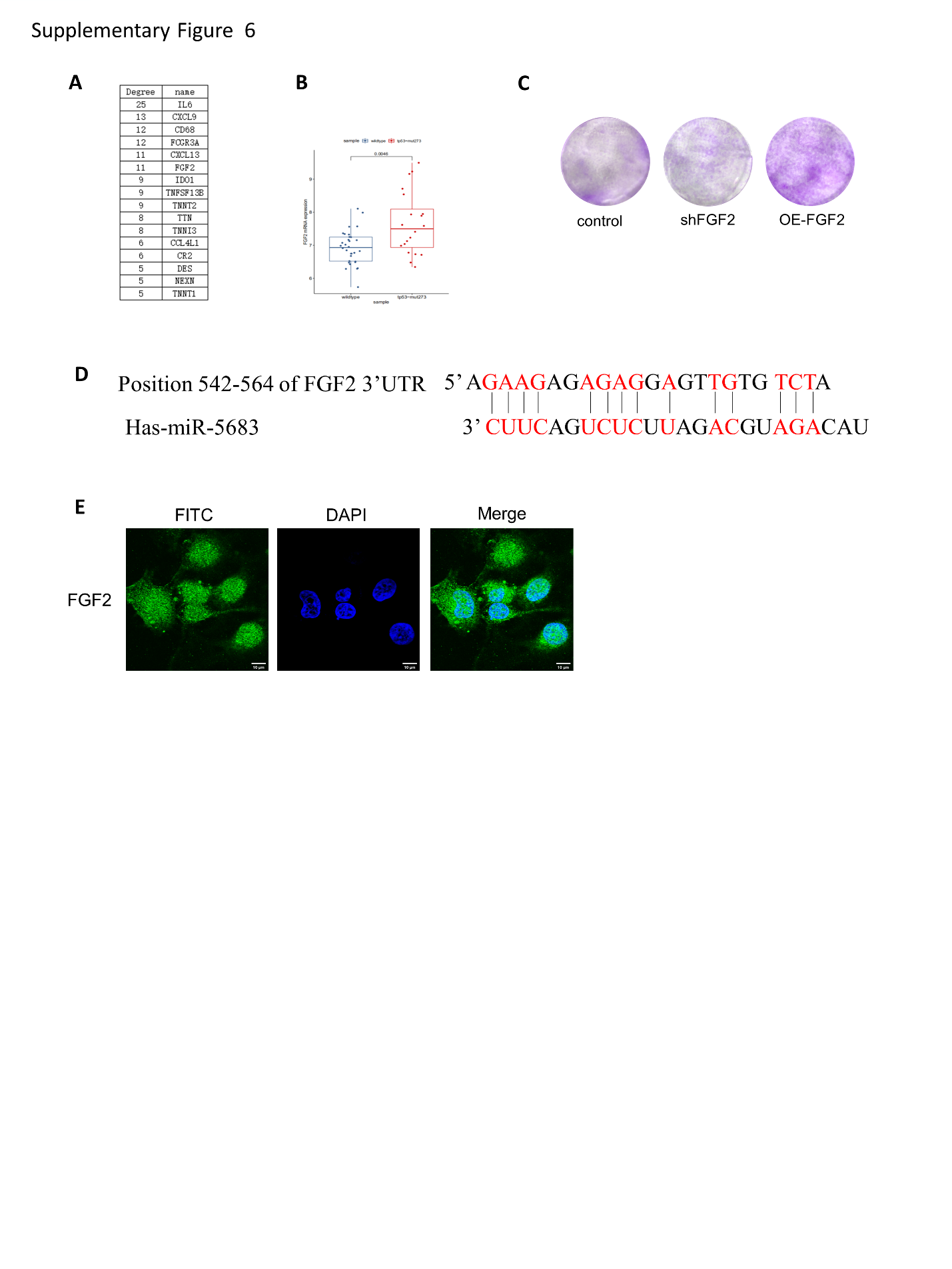
**

**Figure S6**.

(A) The degree value of sixteen core genes. (B) Colony formation of sh*FGF2* and OE-*FGF2* SW480 cells cultured 14 days after crystal violet staining. (C) The predicted binding sites of *miR-5683* in the 3’UTR of *FGF2*. (D) FITC-labeled FGF2 antibody and DAPI were used to observe the subcellular distribution of FGF2 in both the nucleus and cytoplasm of SW480 cells.

**
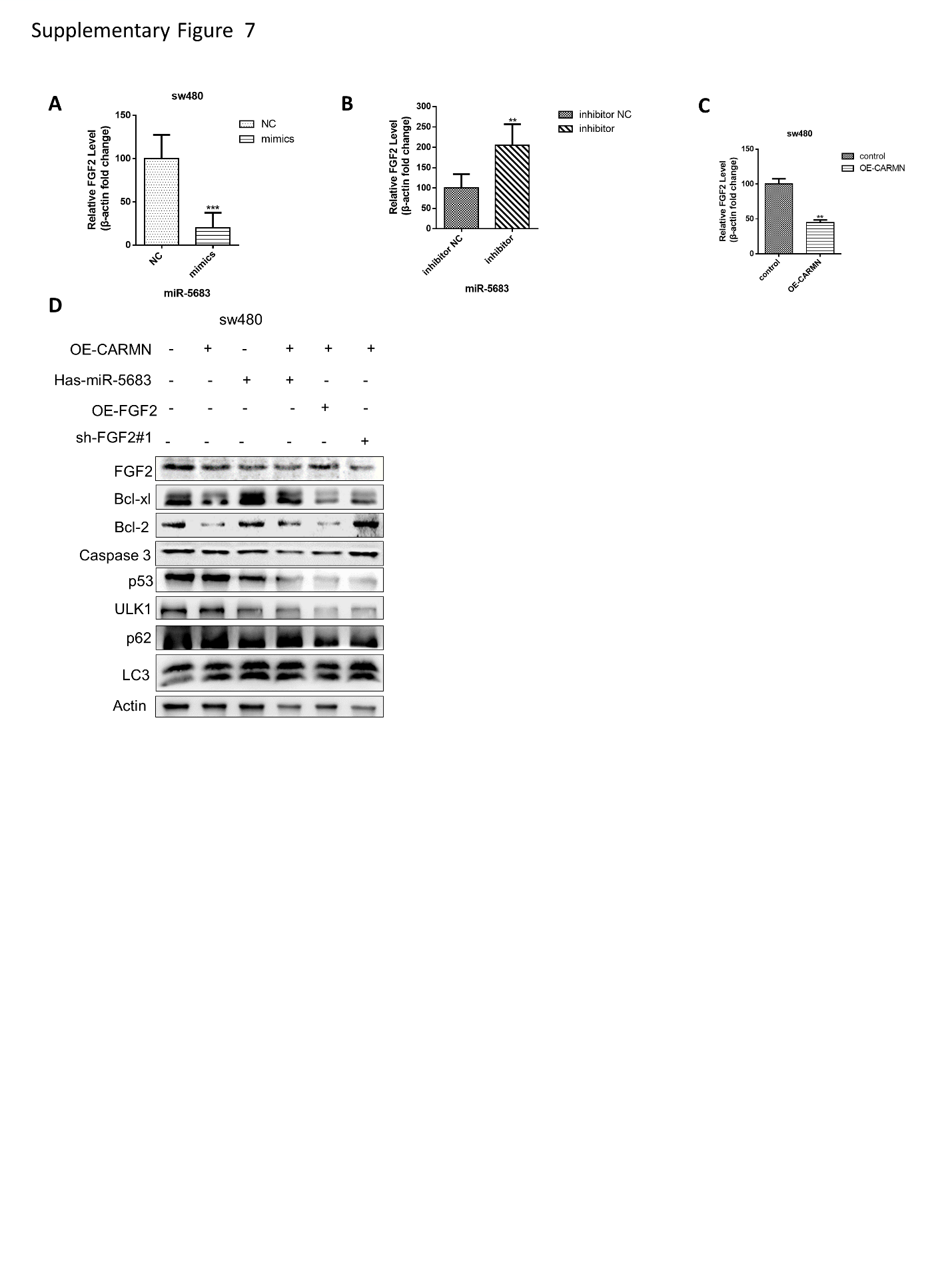
**

**Figure S7.**

(A-C) Expression of *FGF2* was obtained by RT-PCR in SW480 transfected with *miR-5683* mimics, *miR-5683* inhibitors, or OE-*CARMN*. (D) The protein levels of *FGF2, Bcl-xl, Bcl-2, Caspase 3, P53, ULK1, P62*, and *LC3* were detected by western blotting in SW480 cells transfected with OE-*CARMN*, *miR-5683* mimics, OE-*FGF2* or sh*FGF2*.
